# Multidimensional evaluation of four chaihu-shugan-san simulated decoction for use as placebo controls: a comparative study

**DOI:** 10.3389/fphar.2026.1806757

**Published:** 2026-08-04

**Authors:** Kaiyue Huang, Ting Zheng, Jiayan Li, Mi Lv, Xudong Tang, Fengyun Wang, Lin Lv

**Affiliations:** 1 Institute of Digestive Diseases, Xiyuan Hospital of China Academy of Chinese, Medical Sciences, Beijing, China; 2 Graduate School, China Academy of Chinese Medical Sciences, Beijing, China

**Keywords:** botanical drug, chaihu-shugan-san (CSS), evaluation, placebo, sensory instrument

## Abstract

**Background and aim:**

Currently, the preparation and evaluation of botanical placebos for decoctions lack standardization. This study conducted a multidimensional evaluation of an inert excipient decoction placebo for Chaihu-Shugan-San (CSS) and three low-dose CSS formulations, to determine the optimal CSS formulation for use in the control group of clinical randomized controlled trials. The Four Pillars of Best Practice in Ethnopharmacology guided the analysis.

**Materials and Methods:**

CSS was diluted 40-fold, 20-fold, and 10-fold, according to traditional simulated decoction production methods, to obtain 2.5% CSS, 5% CSS, and 10% CSS formulations. These were evaluated together with a pure excipient placebo (pure excipient PBO), consisting solely of excipients and additives. Four CSS decoction formulations were compared for color, odor, and taste using a human scoring method (Likert scale, n = 30). Along with intelligent sensory tools such as a colorimeter, electronic nose, and electronic tongue. Concurrently, the potential pharmacological effects of 4 CSS decoction formulations were examined in animal experiments, including gastric emptying and small intestinal propulsion (n = 80), hot plate analgesia, and the xylene ear swelling test (n = 49).

**Results:**

Manual scoring method indicated that the 10% CSS, 5% CSS and 2.5% CSS (difference, 1.57, 1.26 and 0.96 points, respectively, *P* < 0.01, power = 0.95) scored higher than the pure excipient PBO, with no significant difference in similarity between 5% CSS and 10% CSS (*P* > 0.05, power = 0.95). The intelligent sensory instrument results revealed that pure excipient PBO displayed the closest color match to the CSS decoction (*ΔE* = 2.05), while 10% CSS most closely approximated the CSS decoction in odor. Taste differences between the 4 CSS formulations and CSS were significant. Pharmacological studies found no statistically significant differences between the 4 CSS formulation groups and the control group in gastric emptying rate or small intestine propulsive motility (*P* > 0.05, power = 0.95) nor in ear swelling intensity (*H* = 8.935, *P* = 0.177) or pain threshold (*P* > 0.05, power = 0.004).

**Conclusion:**

10% CSS and 5% CSS, while most similar in appearance to the CSS decoction, did not show superior in improving gastrointestinal motility, anti-inflammatory properties, or analgesic efficacy. However, their safety profile still requires future validation through animal models and quantitative mass spectrometry analysis.

## Graphical Abstract

**Figure:**
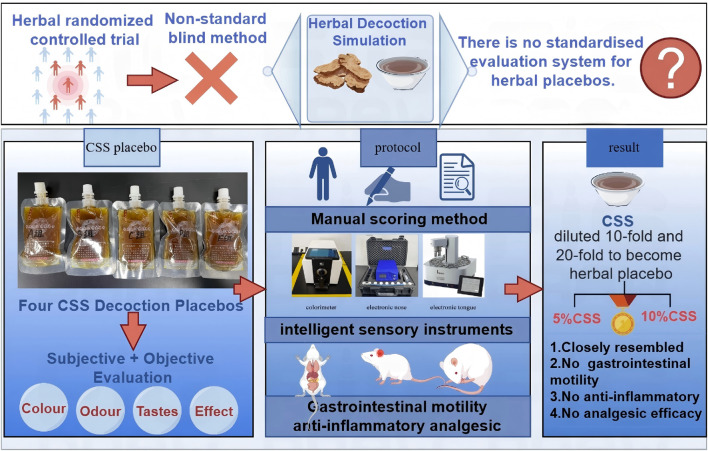

## 1 Introduction

The World Health Organization (WHO) has explicitly declared that traditional, complementary and alternative medicine plays a significant role in primary healthcare (WHO, 2025). Global demand for traditional medicine is projected to increase from US$21.381 billion in 2025 to US$35.937 billion by 2032 (Wong et al., 2025). Botanical therapy stands as one of the key kinds of alternative and complementary medicines. To validate its efficacy, a large number of randomized controlled trials (RCTs) have appeared (Tian et al., 2021). However, the majority of botanical drug clinical trials do not assess the color, odor, or taste of botanical placebos and do not use double-blind methods (Huang et al., 2024; Liu et al., 2026; Tian et al., 2021). The study found that 60% of botanical drug studies disclose the composition of placebos, but only 6% of studies conducted quality evaluation of placebos and experimental medicines (Fu et al., 2008). The reason for this is that the composition of the botanical decoction is complex and has special botanical odor and taste, and there is difficulty in odor and taste simulation (Fei et al., 2018). Many RCTs involving botanical drugs fail to reveal placebo composition or relevant details, and in some instances, have not even employed blinding (Liang et al., 2025; Yang et al., 2025). The evaluation system for botanical placebo controls exhibits significant gaps, resulting in low-quality evidence within evidence-based medicine within botanical science. This urgent issue requires resolution.

There are now two main methods for creating botanical decoction placebos. The first involves diluting the investigated medication by a factor of 10–40. The second comprises making the placebo from excipients and additives, without integrating the research medicine (Guo et al., 2022). The predominant method currently employed involves incorporating a specific quantity of the investigational drug, followed by the addition of dextrin and excipients for simulation. For instance, the CSS placebo in the study by Yujiao Wang et al. (Wang et al., 2024) contained 10% CSS, and the Chang’an I Recipe placebo in the study by Xu-dong Tang et al. (Tang et al., 2018) contained 5% investigational drug. A meta-analysis reported that among seven non-capsule botanical placebo formulations, placebos in four studies contained measurable quantities of the investigational drug (Huang et al., 2024). However, in areas such as Japan and South Korea, it is recommended that botanical placebos should not contain the investigated medication (Agency, 2018). Therefore, placebos containing low doses of the study drug do not strictly qualify as placebos. Nevertheless, placebos created exclusively from excipients face technical limitations, and the use of considerable quantities of excipients also poses safety concerns. Current Chinese guidelines approve the practice of incorporating trace amounts of the study drug (China Association of Chinese Medicine, 2022). Consequently, the approach for manufacturing botanical placebos remains disputed, and comparative research on evaluating botanical placebos created by diverse procedures is still absent.

To standardize the design and reporting of placebos, scholars have proposed two significant tools; however, their suitability for botanical placebo trials remains limited. In 2008, Benno Brinkhaus et al. introduced the Placebo Quality Checklist (PQC) (Brinkhaus et al., 2008), emphasizing that researchers should focus on the quality of the placebo itself rather than merely treating it as an inert control. This checklist stresses the importance of ensuring the placebo’s physical characteristics such as appearance, color, odor, and taste closely match those of the trial drug. It also requires evaluation of the placebo’s preparation process, quality control, and the success of blinding. In 2020, Howick’s team further published the TIDieR-Placebo guidelines (Howick et al., 2020), requiring researchers to report in detail on 13 items concerning the placebo or sham control: composition, preparation method, route of administration, background of the administrator, intervention setting, dosage, duration of treatment, compliance, and results of blinding assessments. Both approaches emphasize the need for standardized evaluation. However, in botanical decoction preparations, the formulation’s distinctive odor and taste, coupled with the physical properties of aqueous formulations—such as color, transparency, and sedimentation—changing over storage time, present significant challenges for simulation. Therefore, the current approach to preparing placebo versions of traditional Chinese medicines adheres to the principle of balancing similarity, safety, suitability, and controllability (Guo et al., 2022). Consequently, preparation often relies on diluting the botanical decoction; however, this does not strictly align with the definition of a placebo and remains controversial. Thus, it is particularly important to evaluate these low-dose botanical drug controls. Nevertheless, discrepancies exist between existing guidelines and the evaluation of botanical placebo preparations. Given the difficulty in simulating botanical decoction placebos, research has insufficiently evaluated botanical placebo controls, leading to inadequate rigor in the implementation of blinding botanical medicine-related RCTs. (Huang et al., 2024). Currently, manual scoring methods remain the primary evaluation approach.

CSS originates from the Ming Dynasty medical compendium “*Jingyue Quanshu*,” boasting over 300 years of history and representing a classical botanical formula for treating depression and functional gastrointestinal disorders (Qu et al., 2025; Ren et al., 2026; Wang et al., 2024). Yuebo Jia et al. (Jia et al., 2026) employed the liquid chromatography–tandem mass spectrometry (LC-MS/MS) detection to identify the primary chemical constituents of CSS as flavonoids (24.19%). It primarily exerts its effects through the hypothalamic-pituitary-adrenal (HPA) axis and the gut-brain axis (Guo et al., 2024; Jia et al., 2026; Zhu et al., 2025). Current research demonstrates that CSS enhances gastrointestinal motility in patients with functional dyspepsia, improves gut microbiota composition, and alleviates anxiety and depression (Li L. et al., 2023; Wu et al., 2025; Xu et al., 2025). Therefore, this study proposes to evaluate CSS decoction simulated drugs created by diverse procedures from a multidimensional perspective, incorporating manual scoring, sensory instruments (colorimeters, electronic noses and electronic tongues), and animal experiments. This study evaluated the similarity and pharmacological effects. Fingerprinting, as well as qualitative and quantitative analyses, were performed on CSS-based placebo and three low-dose CSS controls in accordance with the ConPhyMP guidelines (Heinrich et al., 2022). It provides an evaluation approach for botanical placebos and a theoretical foundation and technical assistance for the systematic manufacturing and assessment of botanical placebos in the future.

## 2 Materials and instruments

### 2.1 Botanical drug

The composition of CSS comprises seven botanical drugs, whose botanical names and complete species information have been verified through the Pharmacopoeia of the People’s Republic of China and the http://mpns.kew.org (MPNS) database, as detailed in Table 1 (Wang et al., 2025). All botanical drugs were supplied by the pharmacy department of Xiyuan Hospital, China Academy of Chinese Medical Sciences, and comply with the quality testing standards of the *Chinese Pharmacopoeia*. All botanical drugs are stored at the Xiyuan Hospital of the China Academy of Chinese Medical Sciences, and quality inspection reports are provided in the Supplementary 1.

**TABLE 1:** Complete species and batch number of botanical drugs.

Chinese name	Botanical name	Name in the Chinese pharmacopoeia	Complete species	Part used	Weight, g
Chai Hu	*Bupleurum falcatum* L	Bupleuri radix	Apiaceae	Root	12
Chen Pi	*Citrus reticulata Blanco*	Citri reticulatae pericarpium	Rutaceae	Rind	12
Chuan Xiong	*Conioselinum anthriscoides ‘Chuanxiong'*	Chuanxiong rhizoma	Apiaceae	Root and rhizoma	12
Xiang Fu	*Cyperus rotundus* L	Cyperi rhizoma	Cyperaceae	Root and rhizoma	9
Shao Yao	*Paeonia lactiflora Pall*	Paeoniae radix alba	Paeoniaceae	Root	15
Zhi Qiao	*Citrus × aurantium* L. aurantium	Aurantii fructus	Rutaceae	Fruit	12
Gan Cao	*Glycyrrhiza glabra* L	Glycyrrhizae radix et rhizoma	Fabaceae	Root and rhizoma	6

### 2.2 Excipients and additives

Color corrector: Lemon Yellow (C_16_H_9_N_4_O_9_S_2_ Na_3_, Batch No.: 12,118,139), Shanghai Dye Research Institute Co., Ltd.; Caramel pigment P440 (Batch No.: 20,230,105), Chongqing Yulong Rongtian Food Additives Co., Ltd.

Filler excipients: Dextrin (a partially hydrolyzed product obtained by treating starch with acid, Batch No.: 20,220,912), Jiaxing Bailang Starch Products Co., Ltd.

Flavoring agents: Sucrose octoacetate (C_28_H_38_O_19_, Batch No.: AA220723), Zhejiang Hetang Technology Co., Ltd.; citric acid (Batch No.: TF81220601), Hunan Jiudian Hongyang Pharmaceutical Co., Ltd.; acesulfame (C_4_H_4_KNO_4_S, Batch No.: 23080601N), Nantong Hongxin Chemical Co., Ltd.

Fragrance: Soy sauce (Flavor Code FP90003), sour-flavored powdered flavoring (Flavor Code YP220719), caramel liquid flavor (Flavor Code CC230530), botanical flavoring (Flavor Code CC230618), from Yilun (Shanghai) Flavors and Fragrances Co., Ltd., a Sino-US joint venture; for specific components, see Supplementary 2.

Suspension aid: Carboxymethyl Cellulose Sodium (CMC-Na, C_6_H_7_O_2_(OH)_2_OCH_2_COONa, Batch No.: 180,413), Carbomer (C_3_H_4_O_2_, Batch No.: 0,101,779,969), Shanghai Yunhong Chemical Formulations and Excipients Technology Co., Ltd.

### 2.3 Preparation and composition of CSS and the four types of CSS-simulated decoctions

The CSS solution was prepared by steeping seven botanical drugs from Section 2.1 in sevenfold water (546 mL) for 1.5 h and decocting 2 times, boiling on high heat, and then keeping on low heat for 30 min each time and then filtering to combine two filtrates, which gave us the CSS decoction.

Place 132 mg of carbomer and 132 mg of CMC-Na in a beaker, add 200 mL of purified water, and stir with a magnetic stirrer for 3–4 h to allow them to swell fully. Once completely dissolved, place the mixture in a water bath and heat to 60 °C to obtain the suspension solution. Weigh 80 mg of caramel pigment P440, 2 mg of lemon yellow, 13,000 mg of dextrin, 30 mg of sucrose octoacetate, 8 mg of citric acid, 4 mg of acesulfame, 8 mg of soy sauce, 8 mg of souring agent, 16 mg of caramel liquid flavor and 0.4 mL of botanical flavoring into a beaker. Then, add 200 mL of the suspending agent solution (60 °C), stir thoroughly until dissolved, and obtain the pure excipient CSS placebo.

**FIGURE 1:**
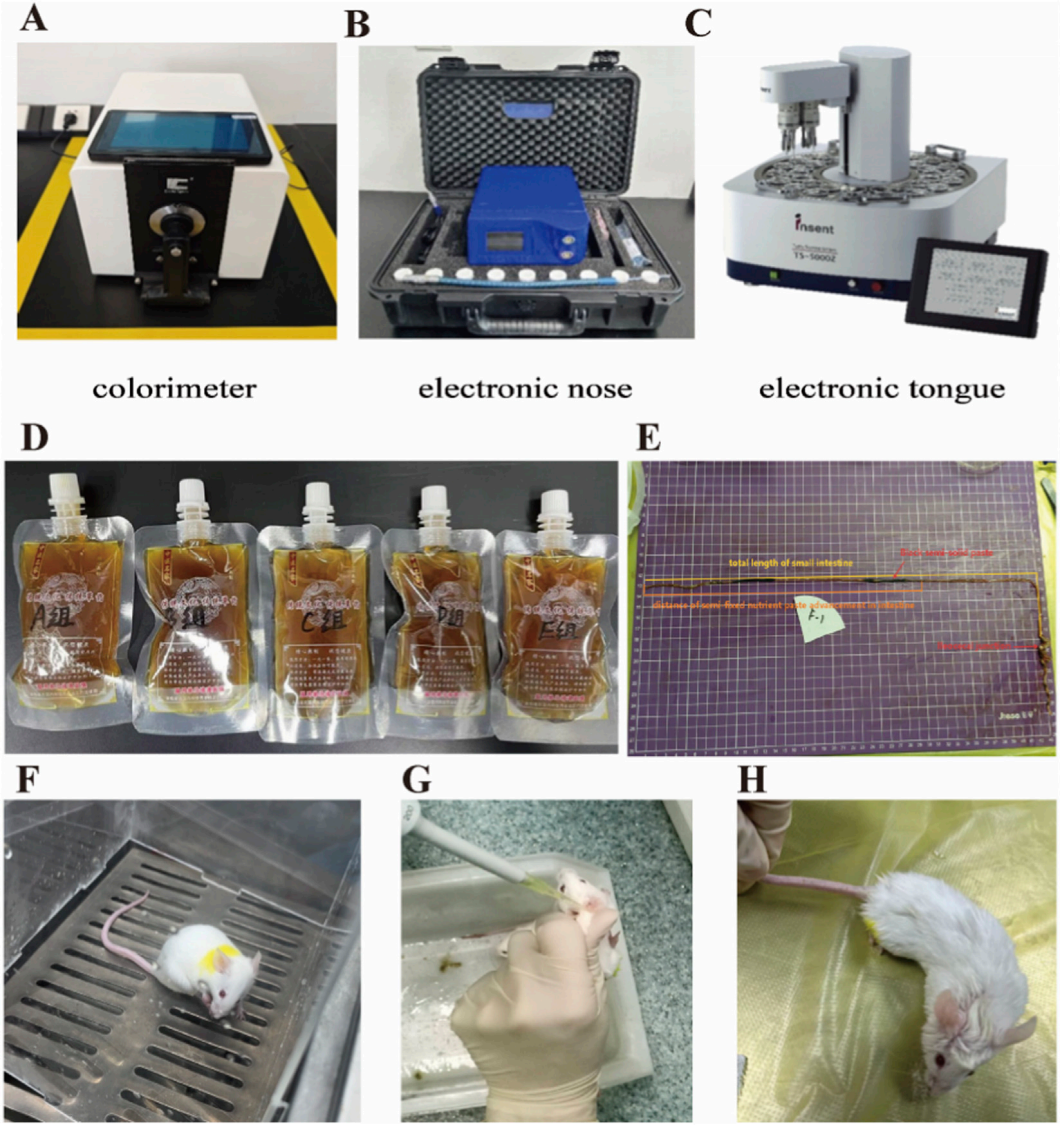
**(A)** colorimeter; **(B)** electronic nose; **(C)** electronic tongue; **(D)** Manual scoring method of CSS-simulated decoctions, during manual evaluation, group names must be concealed and replaced with codes A, B, C, D, and E. Drug **(A)** Bupleurum and Scutellaria Formula (CSS); Drug **(B)** Pure excipient placebo; Drug **(C)** 2.5% CSS containing 2.5% active ingredient; Drug **(D)** 5% CSS containing 5% active ingredient; Drug **(E)** 10%CSS containing 10% active ingredient; **(E)** Semi-solid nutritional paste advancement rate experiment, Semi-fixed nutrient paste advancement rate of the small intestine = distance of semi-fixed nutrient paste advancement in the intestine (cm)/total length of the small intestine (cm) × 100%; **(F)** Foot-licking response in mice; **(G)** Apply xylene to the right ear; **(H)** Swelling in the right ear following application of xylene.

The other three low-dose CSS controls were created by diluting the CSS decoction 10-fold, 20-fold, and 40-fold, respectively, and then adding equal amounts of coloring agent, bittering agent, and dextrin to yield 10% CSS, 5% CSS, and 2.5% CSS (Figure 1A); their compositions are listed in Table 2. To make the 2.5% CSS, combine 5 mL of CSS with 195 mL of pure water, then add 50 mg of caramel pigment P440, 200 mg of dextrin, and 10 mg of sucrose octoacetate, stirring until homogenous. To make 5% CSS, add 10 mL of CSS to 190 mL of pure water, then add 50 mg of caramel pigment P440, 200 mg of dextrin, and 10 mg of sucrose octoacetate, stirring until uniform. To make 10% CSS, add 20 mL of CSS to 180 mL of pure water, then add 50 mg of caramel pigment P440, 200 mg of dextrin, and 10 mg of sucrose octoacetate, stirring until uniform.

**TABLE 2:** Composition of CSS and four types CSS-simulated decoctions.

Drug	Composition (200 mL)
CSS	Chen pi 12 g,Chai Hu 12 g,Chuan Xiong 12 g,Xiang Fu 9 g,Zhi Qiao 12 g,Shao Yao 15 g,Gan Cao 6 g
Excipient PBO	80 mg caramel pigment P440 + 2 mg lemon yellow +13,000 mg dextrin +30 mg sucrose octaacetate +8 mg citric acid +4 mg acesulfame +8 mg soy sauce +8 mg sour +0.16 mL caramel flavor liquid flavor +0.4 mL botanical flavoring + 2 mL CMC-Na + 2 mL carbomer
2.5%CSS	5 mL CSS +195 mL water+50 mg caramel pigment P440 + 200 mg dextrin +10 mg sucrose octaacetate
5%CSS	10 mL CSS +190 mL water +50 mg caramel pigment P440 + 200 mg dextrin +10 mg sucrose octaacetate
10%CSS	20 mL CSS +180 mL water +50 mg caramel pigment P440 + 200 mg dextrin +10 mg sucrose octaacetate

### 2.4 Semi-solid nutritional paste preparation

Take 10 g of sodium carboxymethyl cellulose, dissolve it in 250 mL of distilled water, use a magnetic mixer to mix thoroughly, add 16 g of milk powder, 8 g of sugar, 8 g of starch, and 2 g of activated charcoal powder, respectively, and mix well to formulate a black semi-solid paste of about 300 mL or about 300 g (Duan and Cheng, 2002), and the average weight of 0.3 mL of solid paste was 301 mg.

### 2.5 Instrumentation

#### 2.5.1 Sensory instruments

Benchtop spectrophotometric colorimeter (Model: CS-821N, Hangzhou Color Spectrum Technology Co., Ltd., Figure 1B); electronic nose (Model: PEN3, AIRSENSE, Germany, Figure 1C), which contains 10 different metal oxide sensors to form the sensor array; and electronic tongue (Model: TS-5000Z, Taste Analyzing System, INSENT, Japan, Figure 1D), which is shown in Figures 1A–C.

#### 2.5.2 Liquid chromatography instrument

Waters Acquity UPLC Ultra-High-Performance Liquid Chromatography System—Synapt G2-Si HDMS High-Resolution Ion Mobility Mass Spectrometry Platform (Waters Corporation, USA), Acquity UPLC BEH C18 Column (2.1 mm × 100 mm, 1.7 μm, Waters, USA), Sigma 3–18 K Centrifuge (Sigma, USA) Methanol (chromatographic grade), acetonitrile (chromatographic grade), and methanol (chromatographic grade), acetonitrile (chromatographic grade), and formic acid (chromatographic grade) were all purchased from Fisher; the manufacturers and batch numbers of the reference standards are listed in Supplementary Table S1.

## 3 Methods

### 3.1 Manual scoring method

Ten people were included in each of the doctor, nursing, and patient groups, and the similarity scores of pure excipient PBO, 2.5% CSS, 5% CSS, and 10% CSS were performed according to the principle of single-blindness, with reference to the color, odor and taste of the CSS, and the similarity scoring criterion was based on the internationally accepted Likert scale (Jebb et al., 2021; Qi, 2006). A total score of five points, with higher scores indicating a closer approximation to the CSS, is given in Supplementary Table S2. Before the scoring began, we provided the evaluators with a brief introduction and explanation, covering both the sensory characteristics of the CSS crude drug and a detailed breakdown of the Likert scale. All participants completed their evaluations independently in an environment free from mutual interference.

The cosineθ (cosθ) was used as a metric for evaluating the similarity between the simulated medications and CSS, and if the cosθ value was closer to 1, it indicated a higher degree of similarity between them (Ruo-han et al., 2024). COS is calculated as follows.

$$\cos\theta =\frac{a.b}{\left|\left|a\right|\right|.\left|\left|b\right|\right|}$$

### 3.2 Evaluation of intelligent sensory instruments

#### 3.2.1 Benchtop spectrophotometric colorimeter

Take the sediment-free portion of the botanical sample after full precipitation and stir well to ensure that there are no impurities. Take 15 mL of sample and pour it into a clean and transparent 4.2 × 1.2 × 4.5 cm cuvette without scratches until the height of the cuvette is more than 3/4 full. Select the transmission measurement mode in the operation interface of the instrument, set the light source to D65 and the observer angle to 10°, and complete the calibration of transmission measurement with a standard white board. Place the cuvette smoothly in the transmission test port to ensure that the light path is unobstructed, and then test. *ΔE* is a universal metric for color difference, representing the perceived difference between two colors in a uniform color space. A smaller value indicates a smaller color difference, while a larger value indicates a greater difference. Its calculation formula is as follows:

$$△E=\left[\left(△L*\right){\;}^{2}+\left(△a*\right){\;}^{2}+\left(△b*\right){\;}^{2}\right]{\;}^{0.5}$$

#### 3.2.2 Electronic nose

Take a 10 mL botanical decoction sample in a 100 mL beaker, double-layer with a plastic film seal, and leave it for 1 h. The direct headspace aspiration method was used; the sampling needle was inserted into the sealed beaker, and the electronic nose was measured according to the following parameters: sampling for 1 s/group, self-cleaning of the sensor for 80 s, resetting to zero for 5 s, a sample preparation for 5 s, sample flow rate of 400 mL/min, and analysis of the sampling for 80 s. After the resistance ratio of the sensor has stabilized, the value recorded at the same time shall be selected as the final result.

#### 3.2.3 Electronic tongue

Before measurement, the positive and negative electrodes are immersed in the corresponding cleaning solution for 90 s and then cleaned with the reference solution two times (120 s each time), and the equilibrium test is conducted for 30 s to obtain the potential of the reference solution Vr. The sensor is tested with 35 mL of sample solution for 30 s to obtain the potential Vs. and then calculate the first taste (Vs. - Vr). The sensor was washed in the reference solution for 3 s and then measured the membrane potential Vr’ of the new reference solution (collected for 30 s) and calculated the aftertaste (Vr'-Vr). The data were collected every second, and the 30 s value was taken as the signal output, and the test was repeated 4 times for each sample, and the average value of the last 3 times was taken and analyzed.

### 3.3 UPLC-Q-TOF-MS analytical method for CSS and four CSS-simulated decoctions

#### 3.3.1 Sample preparation

Take 0.2 mL of the test drug, add twice the volume of methanol, and sonicate for 10 min. Centrifuge at 12,000 rpm at 4 °C for 10 min, collect the supernatant, dilute it 10-fold, and inject it into the instrument.

#### 3.3.2 Chromatographic conditions

Acquity Ultra performance liquid chromatography (UPLC) BEH C18 column (2.1 mm × 100 mm, 1.7 μm, Waters, USA); Mobile phase: 0.1% formic acid in water (A) – 0.1% formic acid in acetonitrile (B); Flow rate: 0.3 mL/min; Injection volume: 2.0 μL; Column temperature: 35 °C; Sample compartment temperature: 4 °C; Gradient elution conditions: 0–1.0 min: 95.0% A; 1.0–12.0 min: 95.0%–0% A; 12.0–15.0 min: 0% A; 15.0–15.1 min: 0%–95.0% A; 15.1–17.0 min: 95.0% A.

#### 3.3.3 Mass spectrometry conditions

Electrospray ionization (ESI), data acquisition range m/z 50–1,200. Flow rate: 800 L/h for both positive and negative modes; desolvation gas temperature: 450 °C for positive mode and 400 °C for negative mode; ion source temperature: 110 °C for both positive and negative modes; capillary voltage: 3.5 kV for positive mode and 2.2 kV for negative mode; collision voltage: 30–60 V. Sodium formate was used for mass axis calibration. MassLynx V4.1 was used for data acquisition. Compound identification was performed using Unify V3.6.0.21 in conjunction with published literature.

### 3.4 Laboratory animals and randomized grouping

ICR mice, 22–26 g, 6–8 weeks old, were selected and purchased from Spectrum (Beijing) Biotechnology Co., Ltd., License No.: SCXK (Beijing) 2024-0001. They were bred in the barrier of the animal center of Xiyuan Hospital of the China Academy of Traditional Chinese Medicine, where the environment was simulated to be alternating day and night at 12/12 h, and the constant temperature was maintained at 22 °C–25 °C and 50%–60% relative humidity. The protocol was reviewed by the Ethics Committee of Xiyuan Hospital, China Academy of Traditional Chinese Medicine (2024XLC037-1).

#### 3.4.1 Experiment one (gastric emptying rate and small intestinal propulsion tests)

Based on body weight, 80 ICR mice (equal numbers of males and females) were divided into four district groups, then randomly assigned to eight groups (n = 10/group). These groups comprised the normal control group, CSS group, pure excipient PBO group, 2.5% CSS group, 5% CSS group, 10% CSS group, atropine group (ATP group) and neostigmine group (XSDM group).

#### 3.4.2 Experiment two (hot plate analgesia test and xylene ear swelling experiment)

Based on weight, 49 female ICR mice were divided into seven subgroups and then randomly assigned to seven groups (n = 7/group). These groups comprised the control group, CSS group, pure excipient PBO group, 2.5% CSS group, 5% CSS group, 10% CSS group and aspirin group. As male mice exhibit scrotal descent near the hot plate during thermal pain testing, rendering them sensitive to thermal stimuli, female mice were exclusively employed in this study in accordance with the standard protocols of hot plate analgesia test protocols.

### 3.5 Experimental protocol and dosage regimen

#### 3.5.1 Experiment 1

Following 1 week of adaptive feeding, gastric emptying and small intestinal propulsion experiments were conducted 1 week after dosing in each group. Mouse dose = human dose g/60 kg × 9.1; the dosing regimens for each group were as follows:

Normal control group: pure water (oral gavage)
CSS group: (1,183 g/L, oral gavage)
Pure excipient PBO group (0.2044 g/L, oral gavage)
2.5% CSS group (29.575 g/L, oral gavage)
5% CSS group (59.15 g/L, oral gavage)
10% CSS group (118.3 g/L, oral gavage)
Atropine group (ATP group): (0.1 g/L, intraperitoneal injection)
Neostigmine group (XSDM group): (0.15 mg/kg, intraperitoneal injection)

The dosage for gastric administration is 0.1 mL/g once a day, which is a safe and commonly employed gavage dose for mice weighing 20–30 g. CSS and diluted 2.5% CSS, 5% CSS, and 10% CSS were provided by the preparation room of Xiyuan Hospital, China Academy of Traditional Chinese Medicine. The pure excipient PBO group intervened without the CSS, which was supplied by China Resources Sanjiu Pharmaceutical Co., Ltd. Atropine injection (Henan Runhong Pharmaceutical, Lot No.: 2,404,011) was administered at a concentration of 0.1 g/L; neostigmine mesylate injection (Shanghai Xinyi Jinzhu Pharmaceutical, Lot No.: 2,310,710) was administered at a concentration of 0.15 mg/kg. The above drugs were administered for seven consecutive days.

#### 3.5.2 Experiment 2

The control group, CSS, pure excipient PBO, 2.5% CSS, and 5% CSS medication were the same as part of experiment one; aspirin enteric-coated tablets at a concentration of 3.9 g/L (China Shineway Pharmaceuticals, batch no.: 24,041,011); and all were administered according to the gavage of 0.01 mL/g once a day for seven consecutive days.

### 3.6 Gastric emptying rate

On day 6, fasting and withholding of fluids for 18 h commenced following administration. One hour after the final dose on day 7, 0.3 mL of semi-solid paste was administered *via* gavage. The mice were executed by taking off the cervical vertebrae after 30 min, and the abdominal cavity was opened rapidly, the whole stomach was removed, the whole stomach was swabbed dry with a filter paper and then weighed as the full weight of the stomach, and then the body of the stomach was clipped along the great curvature of the stomach, and the net weight of the stomach was weighed after the stomach was washed and swabbed dry, and the rate of gastric emptying (%) = [1-(full weight of stomach - net weight of stomach)/volume of gastric lavage]×10 (Lin et al., 2021). Dr. Wen, the operator, was unaware of the grouping information and experimental details during the testing.

### 3.7 Small intestinal propulsion experiment

On day 6, fasting and withholding fluids for 18 h commenced following administration. One hour after the final dose on day 7, 0.3 mL of semi-solid paste was administered *via* gavage. 30 min later, off the cervical vertebrae execution of mice, open the abdominal cavity and then quickly separate the mesentery, cut the upper end to the pylorus and cut the lower end of the intestinal tubes to the ileocecal part; pull the small intestine into a straight line and measure from the pylorus to the ileocecal part of the full length of the small intestine and from the pylorus to the black semi-solid paste. The distance from the pylorus to the ileum and the distance from the pylorus to the black semi-solid paste advancement front were measured, and the percentage of the black semi-solid paste advancement front distance to the total length of the small intestine was taken as the small intestine advancement rate, as illustrated in Figure 1E. Semi-fixed nutrient paste advancement rate of the small intestine = distance of semi-fixed nutrient paste advancement in the intestine (cm)/total length of the small intestine (cm) × 100% (Pan et al., 2024). Dr. Fang, the operator, was unaware of the grouping information and experimental details during the testing.

### 3.8 Hot plate analgesia test

Before screening the test and conducting the hot plate analgesia test, set the temperature of the electronic thermostatic water bath to 55 °C. After the temperature is stable, put the mice onto the iron plate and immediately time the mice to lick their feet. The pain threshold time is defined as the duration until the mouse exhibits paw-licking or three or more consecutive frantic jumps. Exclude the mice with the pain threshold of less than 5 s or greater than 30 s and the cut-off time of 60 s. Exclude the oversensitive and unresponsive mice. They were randomly divided into seven groups and administered for 7 days according to the groups. The pre-dose pain threshold was measured (the pain threshold was measured twice, and the average value was taken as the pre-dose pain threshold), and the response time of mice in each group when they started licking their feet (Figure 1F) or jumping frantically more than 3 times was measured 30, 60, 90, and 120 min after the last dose of the drug, respectively.

### 3.9 Xylene ear swelling experiment

1 h after the last administration, use a 100 μL pipette gun to suck 25 μL of xylene in and out of the right ear of the mice (Figures 1G,H), and then take off the neck and execute them after 20 min, then cut off the right and left ears along the auricle of the mice, and then put them on the corresponding labeled filter paper, respectively, and then punch off the ear slices in the same part of the xylene coated with an 8 mm mouse ear puncher, marking the group and the number of the animal, and then weigh them with a 1-millionth scale. Weighing with a one-millionth balance, the degree of swelling and the rate of swelling inhibition were calculated (Yongzhu et al., 2024).

Ear swelling = weight of the right ear - weight of the left ear; Swelling rate = [(Right ear weight - Left ear weight)/Left ear weight] × 100% (Jiaqi et al., 2024).

### 3.10 Statistical methods

SPSS 26.0 statistical software was used for descriptive statistics (standard deviation), and a t-test was used for inferential analyses at a test level of α = 0.05. The normality test was performed first, followed by one-way ANOVA. Tukey HSD is employed if variances are homogeneous; Tamhane’s T^2^ method was employed when variances were heterogeneous. Failure to conform to normal distribution was tested by a non-parametric test, and P < 0.05 indicated that the difference was statistically significant. Then use the PASS 2025 software to calculate the statistical power. For manually scored data, the Kendall’s coefficient of concordance was used to assess inter-rater reliability.

## 4 Results

### 4.1 Manual scoring indicates that 5% CSS and 10% CSS most closely resemble the CSS decoction

The manual scoring method involved a total of 30 participants, comprising 10 individuals each from the physician group, nursing group, and patient group. Both the physician and patient groups included five males and five females, while 10 female nurses were included in the nursing group (n = 10/group), and the appearance of the five botanical decoctions is shown in Figure 1A. The Kendall’s coefficient test revealed a statistically significant result (χ^2^ = 180.513, p = 0.000 < 0.01, Kendall’s Tau = 0.547), indicating that the scores assigned by the 30 evaluators were consistent and that the reliability was moderate.

#### 4.1.1 The 10% CSS scored highest in color, odor and taste

**FIGURE 2:**
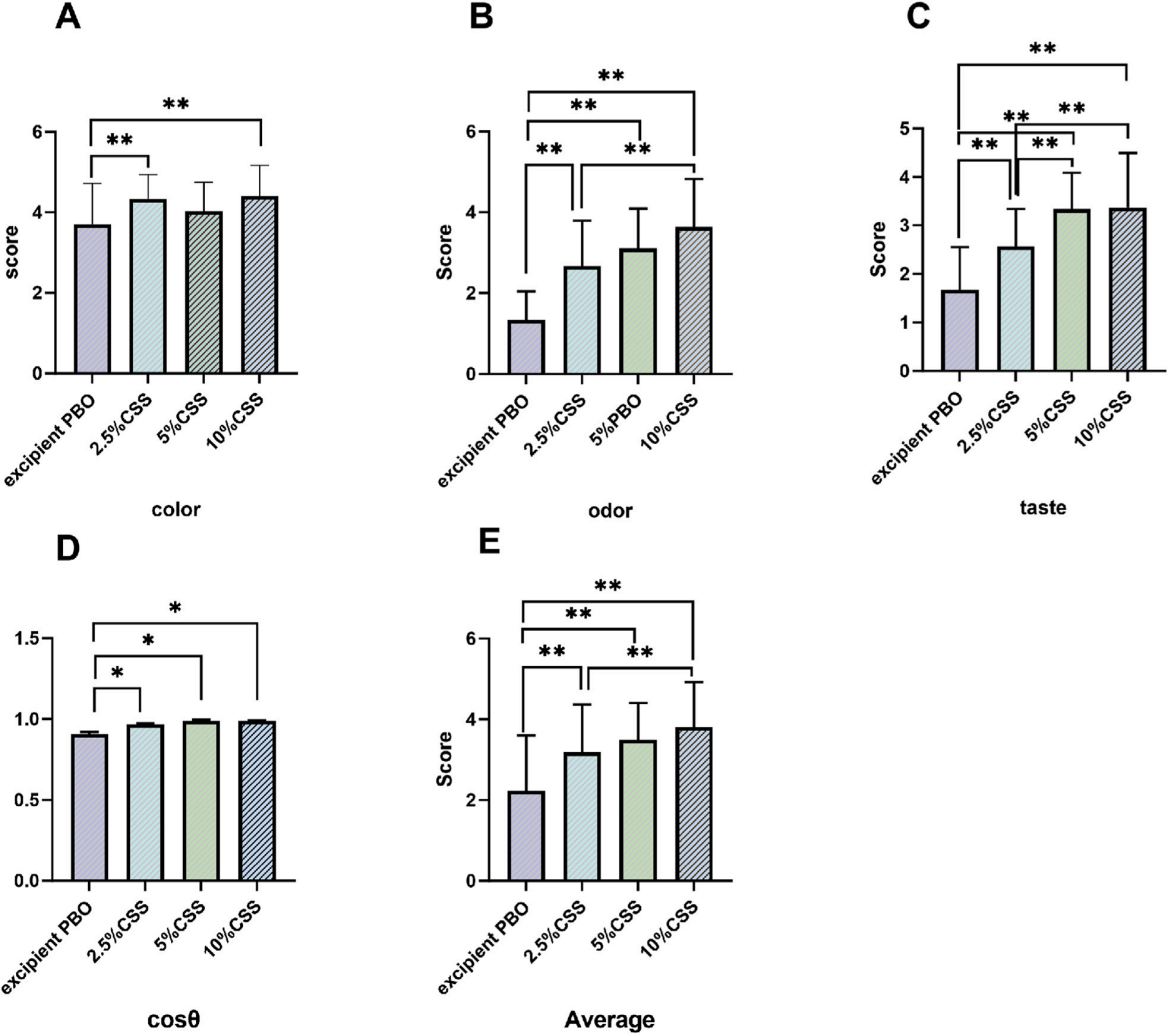
Comprehensive evaluation results of manual scoring methods: **(A)** The average color scores; **(B)** The average odor scores; **(C)** The average taste scores; **(D)** The cosθ similarity values comparing each CSS-simulated decoction group with the CSS reference substance, where the cosθ closer to one indicated a higher degree of similarity; **(E)** The mean total score for each placebo group.

The color scores for pure excipient PBO, 2.5% CSS, 5% CSS, and 10% CSS were 3.70 (1.02), 4.33 (0.61), 4.03 (0.72), and 4.40 (0.77), respectively. The data did not conform to a normal distribution; therefore, the Kruskal–Wallis H test was employed to assess the color scores of the 4 CSS simulated drugs. The test results indicated that the color scores of the four simulated drugs were not entirely identical (*H* = 11.642, *P* = 0.009, power = 0.95). The pairwise comparison results indicated that the 10% CSS achieved the highest color score, significantly exceeding that of the pure excipient group (difference, 0.70 points, *P* = 0.002). However, no significant difference was observed between the 5% CSS and 10% CSS groups (difference, 0.37 points, *P* > 0.05). The pure excipient group recorded the lowest score, significantly lower than both the 5% CSS (difference, 0.33 points, *P* ≤ 0.01) and 10% CSS groups, as illustrated in Figure 2A.

The odor scores for the pure excipient PBO, 2.5% CSS, 5% CSS, and 10% CSS formulations were 1.33 (0.71), 2.67 (1.12), 3.10 (0.99), and 3.63 (1.19), respectively. The data did not conform to a normal distribution; therefore, the Kruskal–Wallis H test was employed to assess the odor scores of the four CSS-simulated decoctions. The test results indicated that the odor scores of the four CSS-simulated decoctions were not entirely identical (*H* = 49.140, *P* < 0.001, power = 0.95). The pairwise comparison results revealed that the 10% CSS achieved the highest odor score, significantly exceeding both the pure excipient group (difference, 2.3 points, *P* < 0.01) and the 2.5% CSS group (difference, 0.96 points, *P* < 0.01). The pure excipient group recorded the lowest score, significantly lower than the 2.5%, 5%, and 10% CSS groups (difference, 1.34, 1.77, and 2.3 points, *P* < 0.01). No significant difference in scores was observed between the 5% and 10% CSS (Figure 2B).

The taste scores for pure excipient PBO, 2.5% CSS, 5% CSS, and 10% CSS were 1.67 (0.88), 2.57 (0.77), 3.33 (0.76), and 3.37 (1.13) respectively. The data did not conform to a normal distribution; therefore, the Kruskal–Wallis H test was employed to assess the taste scores of the four CSS-simulated decoctions. The test results indicated that the taste scores of the four CSS-simulated decoctions were not entirely identical (*H* = 45.662, *P* < 0.001, power = 0.95). The pairwise comparison results revealed that the 10% CSS achieved the highest taste score, significantly exceeding both the pure excipient PBO (difference, 1.7 points, *P* < 0.01)and 2.5% CSS (difference, 0.8 points, *P* < 0.01). The pure excipient PBO recorded the lowest score, significantly lower than the 2.5% CSS, 5% CSS, and 10% CSS groups (*P* < 0.01). No significant difference was observed between the 5% and 10% CSS taste scores. Manual scoring results indicated that the odors of 5% CSS and 10% CSS were closer to CSS than those of 2.5% CSS and the pure excipient group, with the pure excipient group exhibiting the greatest discrepancy (Figure 2C).

#### 4.1.2 Cosθ, a comprehensive evaluation of the similarity of simulated decoctions

The similarity between different CSS-simulated decoctions and the CSS was calculated using cos θ. A cos θ value closer to one indicates greater similarity between the simulated decoctions and the CSS. Compared to the CSS group, the similarity scores for the pure excipient PBO drug were 0.896 for the doctor group, 0.921 for the nursing group, and 0.898 for the patient group; the 2.5% CSS group yielded similarity scores of 0.974, 0.969, and 0.962 for doctors, nurses, and patients, respectively; the 5% CSS group yielded 0.993, 0.984, and 0.994. For the 10% CSS group, the similarity coefficients for the doctor group, nursing group, and patient group were 0.992, 0.989, and 0.986, respectively. The four sets of data conformed to normal distribution with unequal variances. Tamhane’s T2 method was employed, with P < 0.05 indicating statistically significant differences. The results demonstrated that pure excipient PBO had the poorest similarity, which was significantly lower than that of 2.5% CSS (difference, 0.062, *P* < 0.05), 5% CSS (difference, 0.087, *P* < 0.05), and 10% CSS (difference, 0.086, *P* < 0.05); and 5% CSS >10% CSS >2.5% CSS, and there was no significant difference between 5% CSS, 10% CSS, and 2.5% CSS, which were not significantly different from each other in terms of similarity, as illustrated in Figure 2D.

#### 4.1.3 Average score of four simulated decoctions

The pure excipient PBO had color, taste, and odor scores of 3.70 (1.02), 1.33 (0.71), and 1.67 (0.88), with an overall average of 2.23 (1.37) points. The 2.5% CSS had color, taste, and odor scores of 4.33 (0.61), 2.67 (1.12), and 2.57 (0.77), with an average score of 3.19 (1.18). The color, taste, and odor scores for 5% CSS were 4.03 (0.72), 3.10 (0.99), and 3.33 (0.76), with an average score of 3.49 (0.91); for 10% CSS, the values were 4.40 (0.77), 3.63 (1.19), and 3.37 (1.13), with an average score of 3.80 (1.12). The mean score data did not conform to a normal distribution; therefore, the Kruskal–Wallis H test was employed to analyze it. The test results indicated that the mean scores of the four CSS-simulated decoctions differed significantly (*H* = 66.636, *P* < 0.01, power = 0.95). In summary, the 10% CSS had the highest mean score, which was significantly higher than that of the pure excipient PBO (difference, 1.57 points, *P* < 0.01)and 2.5% CSS (difference, 0.61 points, *P* < 0.05), and there was no significant difference with 5% CSS (difference, 0.31, *P* = 0.061); the pure excipient PBO had the lowest mean value, and the statistical results indicated that it was significantly lower than that of the other three groups, and there was no significant difference between the 2.5% CSS and the 5% CSS, and the comprehensive evaluation of 5% CSS and 10% CSS was closer to the CSS decoction, as shown in Figure 2E and Supplementary Table S3.

### 4.2 Intelligent sensory instruments

#### 4.2.1 The color of pure excipients PBO most closely resembles the CSS decoction

**FIGURE 3:**
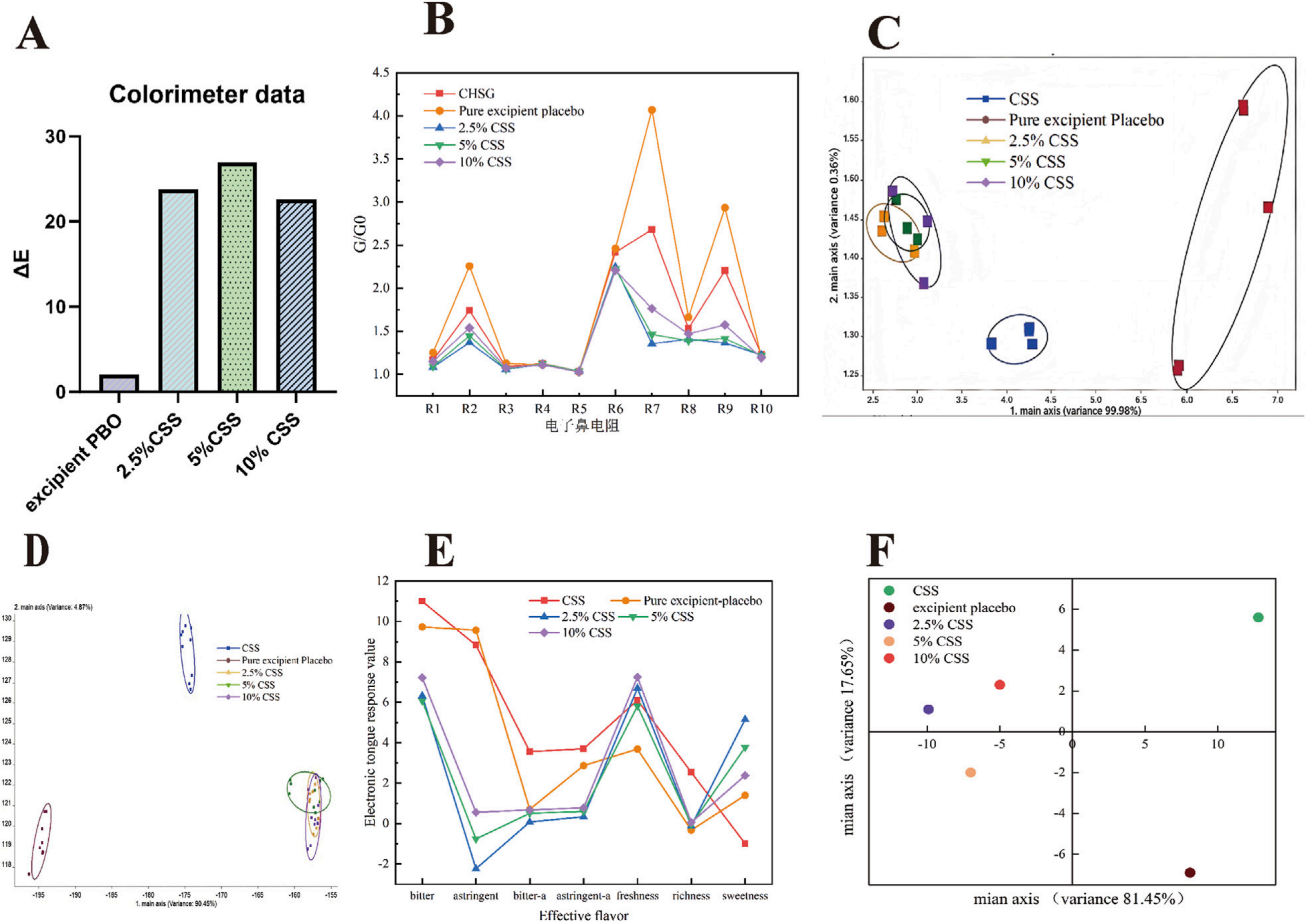
**(A)** Colorimeter data for CSS-simulated decoction; **(B)** Response data for CSS and four CSS-simulated decoctions electronic nose resistors; **(C)** PCA results for the electronic nose: the sum of the contribution rates for the first and second principal components in PCA approaches 99%, with the first principal component accounting for 99.28% and the second principal component accounting for 0.37863%. Combined with the PCA discrimination analysis table (derived from the electronic nose software’s internal calculations, where values closer to one indicate more pronounced sample differentiation), the results show 10% CSS > pure excipient PBO >2.5% CSS >5% CSS. The 2.5% CSS, 5% CSS, and 10% CSS samples are closely matched in terms of odor, with the most pronounced difference observed between the pure excipient PBO and the other samples. **(D)** LDA analysis and contribution rate table for CSS and four CSS-simulated decoctions: among these, the odours of 2.5% CSS, 5% CSS, and 10% CSS are very similar. The greatest difference is observed between pure excipient PBO and the other samples, followed by CSS. In terms of distance, 2.5% CSS, 5% CSS, and 10% CSS are closer to CSS. **(E)** Electronic tongue effective taste response curves for CSS and four CSS-simulated decoction; **(F)** PCA results for the electronic tongue: the electronic tongue PCA plot displays principal components 1 and 2 as the horizontal and vertical axes respectively, with variance contribution rates of 81.447% and 17.654%. Based on the valid taste indicators from this test, a cluster analysis was performed on the five samples, with results shown in Figure 3F. In terms of overall taste profile, the five samples are clearly distinguishable. The figure indicates the greatest divergence between CSS and the other samples. CSS is relatively close to the pure excipient PBO on the first principal component, with both CSS and the pure excipient PBO positioned in the positive direction of the X-axis. Meanwhile, 2.5% CSS, 5% CSS, and 10% CSS are situated on the negative axis of the X-axis, with the three samples being relatively close to one another.

Following measurement by a colorimeter, the L (luminance), a (red-green value), b (yellow-green value), c (saturation), and h (hue angle) values for CSS were determined to be 70.29, 6.60, 68.25, 68.57, and 84.49 respectively. These values were used to calculate the delta-E (*ΔE*) color difference between the CSS-simulated decoctions and CSS and the specific data of the colorimeter are shown in Supplementary Table S4. The smaller *ΔE*, the closer it is to the comparison object: *ΔE* pure excipient PBO (2.05) < *ΔE* 10% CSS (22.60) < *ΔE* 2.5% CSS (23.75) < *ΔE* 5% CSS (26.93), and the color of pure excipient PBO is the closest to CSS, followed by 10% CSS, 2.5% CSS, and 5% CSS, as shown in Figure 3A.

#### 4.2.2 The odor of 10% CSS most closely resembles the CSS decoction

##### 4.2.2.1 Resistance change ratio

The raw data of the electronic nose is the ratio of 10 resistance changes G to the resistance change G0 caused by air passing through the sensor, and the attached Figure 3B shows the comparison of G/G0 values of five samples, and multiple sensors of the electronic nose are more sensitive to the botanical decoction, and the ones with larger and good response values are No. 7, No. 6, No. 9, No. 2, and No. 8, and the response values are large and good, and there is a difference between five samples in terms of response value magnitude and some differences; the original spectrum of the electronic nose is shown in Supplementary Table S1.

##### 4.2.2.2 Principal component analysis (PCA) of electronic nose

The combined contribution rate of the first and second principal components in PCA approaches 99%, with the first principal component accounting for 99.28% and the second principal component accounting for 0.39%. Combined with the PCA discrimination analysis table (where values closer to one indicate more pronounced sample differentiation, Supplementary Table S5). The results of PCA analysis of e-nose Figure 3C show the similarity of odor of simulated decoction compared to CSS: 10% CSS > pure excipient PBO >2.5% CSS >5% CSS. 2.5% CSS, 5% CSS, and 10% CSS are close odor-wise, with the greatest difference between the pure excipient PBO and the other samples.

##### 4.2.2.3 Linear discriminant analysis (LDA) of electronic nose

The LDA analysis results are shown in Figure 3D, where the distinction between CSS and the four CSS-simulated decoctions is more pronounced. Among these, the odors of 2.5% CSS, 5% CSS, and 10% CSS are very similar. The greatest difference is observed between pure excipient PBO and the other samples, followed by CSS. In terms of distance, 2.5% CSS, 5% CSS, and 10% CSS are closer to CSS.

#### 4.2.3 All four CSS-simulated decoctions tastes dramatically different from CSS

##### 4.2.3.1 Electronic tongue data

Using the output of the reference solution as the zero point, the taste items greater than the tasteless point were used as the effective taste evaluation of the samples, except for sour and salty. Bitter, astringent, bitter aftertaste, astringent aftertaste, freshness, richness, and sweetness exceeded the tasteless point, and the specific results are shown in Supplementary Table S6 and Figure 3E.

##### 4.2.3.2 PCA of electronic tongue

The electronic tongue PCA plot displays principal components 1 and 2 as the horizontal and vertical axes respectively, with variance contribution rates of 81.447% and 17.654%. Based on the valid taste indicators from this test, a cluster analysis was performed on the five samples, with results shown in Figure 3F. In terms of overall taste profile, the five samples are clearly distinguishable. The figure indicates the greatest divergence between CSS and the other samples. CSS is relatively close to the pure excipient PBO on the first principal component, with both CSS and the pure excipient PBO positioned in the positive direction of the X-axis. Meanwhile, 2.5% CSS, 5% CSS, and 10% CSS are situated on the negative axis of the X-axis, with the three samples being relatively close to one another.

### 4.3 Determination of the relative concentration of CSS components by UPLC-Q-TOF-MS

Referring to relevant studies on CSS (Jia et al., 2026; Zhang et al., 2024), 29 major components of CSS and CSS-simulated decoctions were analyzed using UPLC-Q-TOF-MS for qualitative fingerprinting and relative quantification. The results showed that no major CSS components were detected in the pure excipient PBO. The total ion intensity of the major components in the 2.5% CSS, 5% CSS, and 10% CSS samples decreased by 82.20%, 71.06%, and 58.91%, respectively, compared to that in CSS. It should be noted that because time-of-flight mass spectrometry was used to measure the peak areas of the major components, signal saturation occurred for some compounds with strong signals in the CSS and low-dose CSS control samples. Consequently, the changes in peak area did not exhibit a linear relationship with concentration changes, but a clear downward trend was observed. For specific data, please refer to supplementary 2; for the ion chromatogram, please refer to Supplementary Table S2. Therefore, a triple quadrupole Multiple Reaction Monitoring (MRM) method was employed to target and quantify liquiritin, saikosaponin D, and albiflorin. Linearity was verified, and the CSS values as well as the concentrations of the three quantified compounds in samples from different treatment groups are presented in Supplementary 3.

### 4.4 Pharmacodynamic experiments

#### 4.4.1 Four types of CSS-simulated decoctions showed no improvement in gastric emptying rate

**FIGURE 4:**
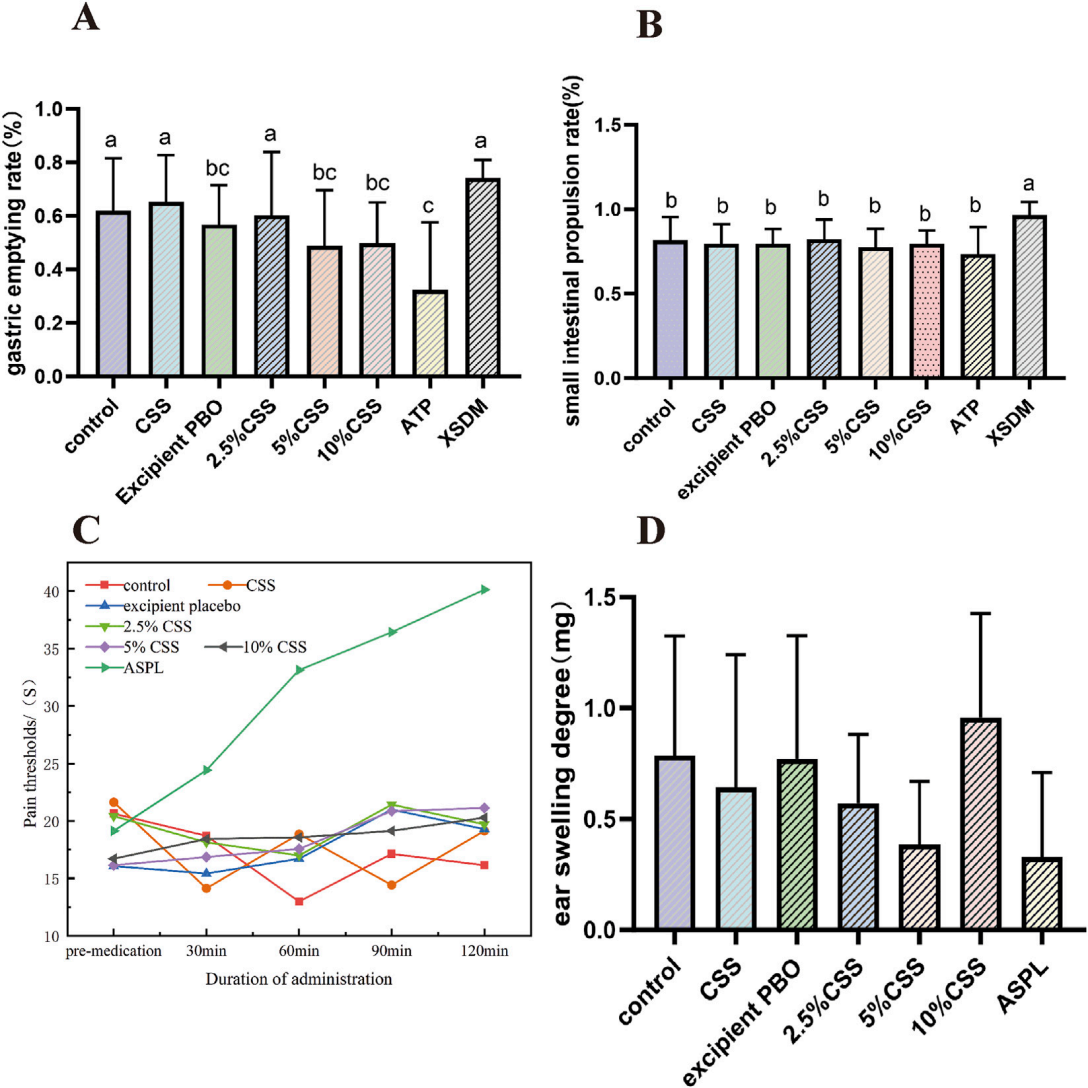
**(A)** Gastric emptying rate: There was a significant difference between the ATP group and the control group, CSS, 2.5% CSS, XSDM, and pure excipient PBO groups, and there was no significant difference in gastric emptying rate between the pure excipient PBO, 2.5% CSS, 5% CSS, 10% CSS groups, and the control group (*P* > 0.05); **(B)** small intestinal propulsion rate: There was a significant difference in small intestinal propulsion rates between the XSDM group and the control group, the CSS, the pure excipients PBO, 2.5% CSS, 5% CSS, 10% CSS, and the ATP group (*P* = 0.005). There was no significant difference in small intestinal propulsion rate between the CSS-simulated decoctions group, pure excipient PBO, 2.5% CSS, 5% CSS, 10% CSS group, and control group (*P* > 0.05). **(C)** Line graph of threshold change for hot plate analgesia test: Pain thresholds at 30, 60, 90 and 120 min post-administration showed no significant difference between the pain thresholds of the control group, the CSS, the pure excipient PBO, the 2.5% CSS, the 5% CSS group, and the 10% CSS group after the administration of the drug compared with that of the pre-administration group, but were significantly lower than those in the ASPL. **(D)** Degree of swelling and swelling inhibition in xylene test ears: There were no significant differences in ear swelling among the groups.

The gastric emptying rate for each group was illustrated in Figure 4A and Supplementary Table S7. The control group’s gastric emptying rate was 62.06% (0.20), while the CSS group’s was 65.28% (0.17). The gastric emptying rate varied by group: pure excipient PBO (56.68% ± 0.15), 2.5% CSS (60.40% ± 0.24), 5% CSS (48.84% ± 0.21), 10% CSS (49.83% ± 0.15), ATP (32.19% ± 0.26), and XSDM (74.12% ± 0.07). Gastric emptying rate data did not conform to normal distribution; the non-parametric Kruskal–Wallis H test was employed to compare gastric emptying rates across groups. The test results indicated significant differences in gastric emptying rates among the groups (*H* = 22.378, *P* = 0.002, power = 0.95). The ATP group showed significantly lower gastric emptying rates than the control group, CSS, 2.5% CSS, XSDM, and pure excipient PBO groups (difference, −8.38%, −9.03%, −23.41% and −6.05%, respectively. *P* < 0.05). Conversely, the XSDM group demonstrated significantly higher gastric emptying rates than the ATP group, pure excipient PBO group, 5% CSS group, and 10% CSS group (*P* < 0.05). The results indicated that there was no significant difference in gastric emptying rate between the pure excipient PBO, 2.5% CSS, 5% CSS, 10% CSS and the control group (*P* > 0.05).

#### 4.4.2 Four types of CSS-simulated decoctions showed no improvement in the semi-fixed nutrient paste advancement rate of the small intestine

The small intestinal propulsion rate varied by group: control (81.65% ± 0.14), CSS (79.47% ± 0.12), pure excipient PBO (79.32% ± 0.09), 2.5% CSS (82.30% ± 0.12), 5% CSS (77.52% ± 0.11), 10% CSS (79.44% ± 0.08), ATP (73.27% ± 0.16), and XSDM (96.68% ± 0.08), as detailed in Figure 4B and Supplementary Table S7. Data did not conform to normal distribution; therefore, the non-parametric Kruskal–Wallis H test was employed, revealing that the small intestinal propulsion rate in the XSDM group was significantly higher than those in the control group, the CSS, the pure excipients PBO, 2.5% CSS, 5% CSS, 10% CSS, and the ATP groups (difference, 15.03%, 17.21%, 17.36%, 14.35%, 19.16%, 17.24% and 23.41%, respectively. *H* = 20.067, *P* = 0.005, power = 0.95). There was no significant difference in small intestinal propulsion rate between the simulated decoctions group, pure excipient PBO, 2.5% CSS, 5% CSS, 10% CSS group, and control group (difference, −2.33%, 0.65%, −4.13% and −2.21%, respectively. *P* > 0.05).

#### 4.4.3 Four types of CSS simulated decoctions showed no pharmacological effect in raising pain thresholds

The pain thresholds were measured after 30 min, 60 min, 90 min, and 120 min after the last administration, respectively. Data from the control group, CSS group, pure excipient group, and 5% CSS group did not conform to normal distribution; non-parametric Kruskal–Wallis H analysis was employed. The 2.5% CSS, 10% CSS, and ASPL groups exhibited normal distribution with homogeneity of variance; ANOVA-LSD analysis was applied. Intragroup analysis showed that there was no significant difference between the pain thresholds of the control group (*H* = 3.341, *P* = 0.502), the CSS (*H* = 6.812, *P* = 0.146), the pure excipient PBO (*H* = 2.778, *P* = 0.596), the 2.5% CSS (*F* = 0.393, *P* = 0.812), the 5% CSS group (*H* = 6.280, *P* = 0.179), and the 10% CSS group (*F* = 0.220, *P* = 0.925) after the administration of the drug compared with that of the pre-administration group. There was no significant increase in the pain threshold at 30 min after administration in the ASPL group (difference, 5.286 s, 95% *CI*: 4.674 to 15.246, *P* = 0.546), and there was a significant increase in the pain threshold at 60 min (difference, 14.000 s, 95% *CI*: 4.040 to 23.960, *P* = 0.003), 90 min (difference, 17.286 s, 95% *CI*: 7.326 to 27.246, *P* = 0.000), and 120 min (difference, 21.000 s, 95%*CI*: 11.040 to 30.959, *P* = 0.000) after administration compared with that before administration.

In intergroup comparisons, data across all time points failed to meet normality assumptions. The Kruskal–Wallis H test was employed to assess pain thresholds at each time point. There was no significant difference in pain thresholds between the five groups’ pre-administration pain thresholds (*P* = 0.312) or 30 min post-administration pain thresholds in each group (*P* = 0.067). However, significant differences were observed in pain thresholds at 60 min (*H* = 17.808, *P* = 0.007), 90 min (*H* = 17.805, *P* = 0.007), and 120 min (*H* = 18.565, *P* = 0.005) across the five groups. Post-hoc analysis revealed that pain thresholds in the five groups were all significantly lower than the ASPL group (*P* < 0.05), as shown in Supplementary Table S8 and Figure 4C.

#### 4.4.4 Four types of CSS-simulated decoctions showed no improvement in ear swelling

Compared with the control group, only 10% of the PBO group experienced more severe swelling, and the 10% CSS group did not inhibit swelling relative to the control group, and the ear swelling degree of each group is shown in Figure 4D and Supplementary Table S9. Compared with the control group, the swelling inhibition rate of ASPL (58.23%)>5% CSS (50.63%)>2.5% CSS (27.85%)>CSS (18.99%)>pure excipient PBO (2.53%)>10% CSS (−21.52%), but statistical analysis results showed that there was no significant difference in the degree of swelling between the groups (*H* = 8.935, *P* = 0.177, power = 0.004).

## 5 Discussion

This study evaluated CSS-simulated decoctions prepared by four distinct methods using manual scoring method, intelligent sensory instrumentation, and pharmacodynamic experiments. Additionally, triple chemical fingerprinting was used to measure the peak area ratios of CSS and the major components of CSS-simulated decoctions. Manual scoring results indicated that 5% and 10% CSS most closely resembled the CSS decoction and were significantly superior to the pure excipient placebo (P < 0.01). Sensory instrument testing further revealed that the pure excipient placebo most closely matched the CSS in color (*ΔE* = 2.05), while the 10% CSS most closely matched the CSS in odor. However, the taste of all four CSS-simulated decoctions differed significantly from the CSS. Pharmacodynamic studies found no significant differences in gastric emptying rate, small intestinal propulsion rate, pain threshold changes, or ear swelling degree between the four CSS-simulated decoction groups and the control group (*P* > 0.05). Whereas the positive drug groups showed significant pharmacological effects (P < 0.01). Nevertheless, data on xylene expansion are scattered and inconsistent, and the statistical validity is insufficient. The concentrations of the major chemical components in the low-dose CSS controls show a significant decreasing trend with increasing dilution ratio; however, due to signal saturation in some compounds with strong signals, the relationship between peak area and concentration is not linear. Strictly speaking, the three low-dose CSS controls are not placebos, but they are commonly used in RCTs involving decoctions of botanical drugs. However, since this study aims to advance the preparation and evaluation of placebos for decoctions of botanical drugs, the discussion here focuses on the requirements for placebos. Consistency of placebos in packaging, color, odor, and taste is the basic requirement for the implementation of blinding (Yuan et al., 2025). Additionally, it must also be ensured that the placebo possesses no therapeutic effect whatsoever, which is the role of placebos in scientific experiments (Dobrilla and Scarpignato, 1994). Preliminary pharmacodynamic animal tests reveal that none of the four CSS-simulated decoctions exhibit pharmacological benefits by increasing gastrointestinal motility or exhibiting anti-inflammatory and analgesic qualities. However, all four CSS-simulated decoction versions displayed major taste changes from the CSS decoction. Pure excipient PBO is made entirely from excipients and does not contain CSS prodrugs, all of which have a wide variety of excipients, but its similarity is still significantly lower than that of 2.5% CSS, 5% CSS, and 10% CSS, which is thought to be a result of the insufficient simulation technology of the current placebo simulation of botanical drugs through analysis. Although there are many Chinese medicine placebos nowadays simulated by using pure excipients, and the color aspect can almost reach the consistency, due to the specificity of the smell and taste of the Chinese medicine compound, using a variety of tastes and orthodontics to simulate it, the compound taste will be more pungent, and it is still challenging to achieve the same level as that of the original medicine. In contrast, 2.5% CSS, 5% CSS, and 10% CSS were added with 2.5%, 5%, and 10% of the CSS decoction, respectively, and the types and amounts of excipients added were reduced, and the color, odor, and taste were lighter than CSS, but the similarity was higher.

Currently, research on botanical placebos for decoctions is sparse. In 2007, the University of the Western Cape, Bellville, employed step-wise solvent extractions to create a placebo for the Artemisia afra botanical drug. Linalool and sodium saccharin were used to simulate its scent and taste. Pharmacological observations were undertaken utilizing guinea-pig trachea to examine if the botanical concoction and placebo possessed calming activity. The final results demonstrated that color simulation proved pretty good; nevertheless, the placebo manufactured utilizing chemical additives exhibited a considerable disparity in both scent and taste compared to the Artemisia afra botanical drug (Dube A, et al., 2007). This is highly consistent with our findings that pure excipient placebos exhibit high color matching but low odor and taste matching. Zhang et al. analyzed WHO-registered clinical trials of traditional Chinese medicine and found that 97.4% (75/77) of studies did not evaluate the placebo (Qi GD, et al., 2008). Li et al.'s cross-sectional analysis of clinical trials for rare disease Chinese botanical drugs revealed that 18.2% of placebos contained active ingredients, yet none of the studies conducted pharmacologically inert tests (Li Y. et al., 2023). All studies indicate that the distinctive odors and tastes of botanical compounds significantly increase the difficulty of simulation, particularly for botanical decoctions. Collectively, these investigations reveal a core dilemma within the placebo research of botanical drugs: pure excipient simulations struggle to achieve high similarity, while dilution methods face skepticism regarding their efficacy. The placebo made from pure excipients in our investigation comprised twelve excipients. However, the primary cause for insufficient similarity was the substantial discrepancy in scent and taste, which has repeatedly constituted a bottleneck in simulating botanical drug placebos. Owing to the unique odor of botanical drugs, the use of multiple flavorings and corrective excipients to imitate the compound’s taste results in a more pronounced taste profile, yet it remains difficult to match CSS decoctions. Conversely, by incorporating 2.5%, 5%, and 10% of the CSS decoction, the variety and amount of additives needed were reduced. The resulting product showed lighter color, odor, and taste compared to CSS yet demonstrated higher similarity. This method is frequently employed in the preparation of botanical simulated decoctions through gradual dilution of the botanical drug decoction.

Strictly speaking, the ethical acceptability of placebos is based on the principle of complete inertness. In this study, the low-dose CSS controls do not meet the criteria for complete inertness; therefore, it is inappropriate to refer to them as placebos. If such formulations were used in clinical trials, the presence of trace amounts of active ingredients could introduce bias. However, due to certain unavoidable factors, this approach has persisted. This highlights a dilemma involving a trade-off between the quality of high-blind studies and ethical rigor; the trade-off between high similarity (dilution of active ingredient method) and complete inertness (pure excipient method) remains a subject of ongoing debate. Japan and South Korea recommend that placebos for botanical drugs should contain no investigational drug, emphasizing the gold standard of complete inertness. However, placebos containing less than 10% of the investigational drug are currently accepted in China, and the Food and Drug Administration (FDA) considers drugs containing 90%–110% of the active ingredient to be compliant (ICH, 2021). Based on this, we believe that a placebo in which the detected active ingredient does not exceed 10% of the active ingredient in the CSS and has no pharmacological effect can be considered a compliant simulated drug. This study preliminarily indicates that CSS simulated drugs with 5% and 10% dilution levels showed no detectable significant pharmacological activity in animal experiments, providing some basis for their use under specific circumstances. However, it must be emphasized that negative results from this study alone cannot conclusively assert complete pharmacological inactivity of these simulated decoctions. Li et al.'s research indicates that even when placebos contain active ingredients, the vast majority of studies fail to conduct inertness verification. Although our study conducted qualitative analysis and relative quantification of peak areas for the major components, the signals were too saturated to yield a linear relationship. This highlights the need for future research to conduct quantitative analyses of chemical composition combined with pharmacodynamic validation in disease models to rigorously establish the inertness of placebos.

Therefore, the evaluation process for botanical simulated decoction becomes crucial, as it not only ensures placebo quality but also strives to maintain a balance between high drug similarity (active ingredient dilution method) and complete inertness (pure excipient method). Our evaluation process incorporates assessments using intelligent sensory instruments and pharmacodynamic experiments, rendering it more objective and credible while overcoming the limitations of single-method evaluation. However, the method of preparing placebos using the test drug itself has limitations and risks, and its use should be avoided as much as possible in clinical trials.

## 6 Limitations

First, this study employed healthy animals for pharmacodynamic experiments, which may not fully reveal the effects of trace active components. Normal mice have normal gastrointestinal motility, which is dependent on the brain-gut axis and neural and endocrine regulation (Camilleri, 2021; Singh et al., 2022), and under the physiological condition of the body, the effect of medium-dose CSS in regulating gastrointestinal motility is not obvious, so the difference between the gastric motility and small intestinal advancement of the CSS group and that of the control group is not statistically significant. Second, findings on anti-inflammatory and analgesic effects did not disclose any significant advantage for CSS. The assessment was limited to the hot plate analgesia test and xylene ear swelling test, with no serological indications such as inflammatory factors being investigated. Subsequent studies should use a more comprehensive approach. Third, animal studies lack safety assessments, which should be addressed in future evaluations of botanical placebos.

Simulating botanical decoctions as placebos provides higher obstacles than granules, capsules, or pills, rendering our research both challenging and yet very practical and inventive. Although Artificial Intelligence (AI) prediction and picture recognition are now being proposed for application in the simulation and creation of botanical placebos, their implementation has yet to be totally successful. Utilizing intelligent sensory devices to quantify assessed factors, combined with manual scoring methods and component identification, thereby developing a more comprehensive plant placebo assessment system workflow in the future.

## 7 Conclusion

The production of botanical placebos utilizing pure excipients remains hard in terms of imitating the odor and taste of natural botanical drugs. Simulated decoctions made with pure excipients exhibit lower odor and taste properties compared to those prepared utilizing the diluted decoction combined with the excipient approach. A comprehensive assessment of CSS decoction placebos using the manual scoring method, intelligent sensory instruments and pharmacodynamic experiments revealed that placebos containing 5% and 10% CSS most closely resembled CSS itself, with no evidence of enhanced gastrointestinal motility or anti-inflammatory analgesic effects; however, the statistical power is low, and negative results should be interpreted with caution. The sample size should be increased in subsequent studies to confirm these findings. Current research evaluating botanical placebos is becoming increasingly rigorous, transitioning from empirical to data-driven techniques. In the future, botanical placebo ingredients should undergo component identification, and safety assessments must not be overlooked. For sensory evaluation, integrating artificial intelligence and sensors should be improved to quantify evaluation data and enhance standardization (Fang et al., 2018).

## Data Availability

The raw data supporting the conclusions of this article will be made available by the authors, without undue reservation.
