# Supplementary material for: Multidimensional evaluation of four chaihu-shugan-san simulated decoction for use as placebo controls: a comparative study
Source: Front Pharmacol. 2026 Aug 4;17:1806757. doi: 10.3389/fphar.2026.1806757 (PMC13481859; doi:10.3389/fphar.2026.1806757)
Supplement: Supplementary file 1 [file DataSheet1.pdf]

Table S1 Batch and manufacturer of the reference material

name	Item Number	Manufacturer
Saikosaponin A	RFS-C02401906005	Chengdu Herpurify Co.,Ltd
Albiflorin	CFN99774	Chem Faces
Saikosaponin D	RFS-C02602005022	Chengdu Herpurify Co.,Ltd
Saikosaponin B1	RFS-C08502111026	Chengdu Herpurify Co.,Ltd
Hesperidin	B20182	Shanghai yuanye Bio-Technology Co., Ltd
Senkyunolide H	RFS-Y08402112008	Chengdu Herpurify Co.,Ltd
Senkyunolide A	RFS-Y08301903021	Chengdu Herpurify Co.,Ltd
Ligustrazine	CFN99909	Chem Faces
Senkyunolide I	CFN99596	Chem Faces
Liquiritigenin	YY90042	Shanghai yuanye Bio-Technology Co., Ltd
Liquiritin	B20414	Shanghai yuanye Bio-Technology Co., Ltd
Glycyrrhizic acid	B20417	Shanghai yuanye Bio-Technology Co., Ltd
Paeonol	RFS-D00211812016	Chengdu Herpurify Co.,Ltd
Ligustilide	B20492	Shanghai yuanye Bio-Technology Co., Ltd

Table S2 Manual scoring method form

<table>
<tr><td colspan="7">Name: Gender: Age: Telephone Number:
Group:</td></tr>
<tr><td></td><td></td><td>Almost entirely consistent
(5 points)</td><td>Very close
(4 points)</td><td>Fairly close
(3 points)</td><td>Uncertain
(2 points)</td><td>Completely inconsistent
(1 points)</td></tr>
<tr><td rowspan="3">Drug B</td><td>Color</td><td></td><td></td><td></td><td></td><td></td></tr>
<tr><td>odor</td><td></td><td></td><td></td><td></td><td></td></tr>
<tr><td>taste</td><td></td><td></td><td></td><td></td><td></td></tr>
<tr><td rowspan="3">Drug C</td><td>Color</td><td></td><td></td><td></td><td></td><td></td></tr>
<tr><td>odor</td><td></td><td></td><td></td><td></td><td></td></tr>
<tr><td>taste</td><td></td><td></td><td></td><td></td><td></td></tr>
<tr><td rowspan="3">Drug D</td><td>Color</td><td></td><td></td><td></td><td></td><td></td></tr>
<tr><td>odor</td><td></td><td></td><td></td><td></td><td></td></tr>
<tr><td>taste</td><td></td><td></td><td></td><td></td><td></td></tr>
<tr><td rowspan="3">Drug E</td><td>Color</td><td></td><td></td><td></td><td></td><td></td></tr>
<tr><td>odor</td><td></td><td></td><td></td><td></td><td></td></tr>
<tr><td>taste</td><td></td><td></td><td></td><td></td><td></td></tr>
<tr><td colspan="7">Date:
Physician's Signature:</td></tr>
</table>

Table S3 Comprehensive evaluation results of manual scoring methods (n=30)

	color	odous	taste	Cosθ	average
excipient PBO	3.70 (1.02)	1.33 (0.71)	1.67 (0.88)	0.907	2.23 (1.37)
2.5% CSS	4.33 (0.61)**	2.67 (1.12) ** #	2.57 (0.77) **	0.969*	3.19 (1.18)** #
5% CSS	4.03 (0.72)	3.10 (0.99) **	3.33 (0.76) ** ▲	0.994 *	3.49 (0.91) **
10% CSS	4.40 (0.77) **	3.63 (1.19) **	3.37 (1.13) ** ▲	0.993 *	3.80 (1.12) **

Note: The numbers in parentheses represent the standard deviation. * P < 0.05, vs excipient PBO; ** P < 0.01, vs excipient group; # P < 0.01, vs 10% CSS ;▲ P < 0.01, vs 2.5% CSS.

Table S4 Colorimeter data for CSS simulated decoctions

	L^*	a^*	b^*	c^* （saturation）	h^* （color angle）	ΔE （aberration）
CSS	70.29	6.60	68.25	68.57	84.49	
excipient PBO	68.84	5.36	69.01	69.22	85.56	2.05
2.5% CSS	74.18	4.24	44.94	45.14	84.62	23.75
5% CSS	76.96	2.84	42.43	42.52	86.18	26.93
10% CSS	74.52	3.72	46.23	46.38	85.41	22.60

Table S5 Electronic nose PCA discrimination analysis table

	CSS	pure excipient PBO	2.5% CSS	5% CSS	10% CSS
CSS	/	0.926	0.934	0.937	0.903
pure excipient PBO	0.926	/	0.972	0.972	0.968
2.5% CSS	0.934	0.972	/	0.262	0.333
5% CSS	0.937	0.972	0.262	/	0.092
10% CSS	0.903	0.968	0.092	0.092	/

Table S6 Electronic tongue experiment data for CSS simulated decoctions

	sour	bitter	astringent	bitter-a	astringent-a	freshness	richness	salty	sweetness
tasteless	-13	0	0	0	0	0	0	-6	0
CSS	-15.04	11	8.83	3.56	3.7	6.11	2.54	3	-0.99
excipient-placebo	-7.56	9.73	9.57	0.71	2.87	3.69	-0.32	-6.28	1.4
2.5% CSS	-20.91	6.32	-2.23	0.08	0.34	6.7	-0.12	-14.34	5.16
5% CSS	-16.51	6.08	-0.76	0.51	0.6	5.82	-0.04	-13.94	3.77
10% CSS	-19.93	7.22	0.56	0.68	0.78	7.25	0.06	-10.8	2.38

Note: the bitter-a is bitter aftertaste, the astringent-a is astringent aftertaste.

Table S7 Gastric emptying rate and small intestinal propulsion rate in mice(n=10/group)

group	Gastric emptying rate	small intestinal propulsion rate
control	62.06% (0.20)#	81.65% (0.14)▲
CSS	65.28% (0.17)#	79.47% (0.12)▲
Excipient PBO	56.68% (0.15)▲	79.32% (0.09)▲
2.5% CSS	60.40% (0.24)#	82.30% (0.12)▲
5% CSS	48.84% (0.21)▲▲	77.52% (0.11)▲
10% CSS	49.83% （0.15)▲▲	79.44% （0.08)▲
ATP	32.19% （0.26)*▲▲	73.27% （0.16)▲
XSDM	74.12% (0.21)	96.68% (0.02)

Note: The numbers in parentheses represent the standard deviation. *P≤0.01 vs control group; #P<0.01 vs ATP group; ▲P<0.05 vs XSDM group; ▲▲P≤0.01 vs XSDM group; none of the CSS simulated decoctions groups were significantly different from the blank control group.

Table S8 Threshold changes after administration of hot plate analgesia test (n=7/group)

group	before administration (s)	30 min after administration (s)	60 min after administration (s)	90 min after administration (s)	120 min after administration (s)	*P*
control	20.64 (6.61)	18.71 (11.69)	13.00 (3.92)##	17.14 (8.97)##	16.14 (9.79)##	0.502
CSS	21.64 (5.88)	14.14 (4.14)	18.86 (9.49)##	14.43 (5.59)##	19.14 (9.28)##	0.146
excipient PBO	16.07 (2.26)	15.43 (3.82)	16.71 (6.24)##	21.00 (15.67)##	19.29 (5.22)##	0.596
2.5% CSS	20.43 (8.77)	18.14 (5.15)	17.00 (5.60)##	21.43 (4.65)#	19.71 (4.61)##	0.812
5% CSS	16.14 (3.46)	16.86 (4.10)	17.57 (2.30)#	20.86 (4.49)#	21.14 (5.96)##	0.179
10% CSS	16.71 (6.14)	18.43 (4.39)	18.57 (6.29)#	19.14 (5.18)##	20.29 (3.25)#	0.925
ASPL	19.14 (4.06)	24.43 (5.06)	33.14 (8.09)*	36.43 (6.90)*	40.14 (7.15)*	0.000

Note: The numbers in parentheses represent the standard deviation. *P < 0.01 vs control group; #P < 0.05 vs ASPL; ##P < 0.05 vs ASPL;

Table S9 Degree of swelling and swelling inhibition in xylene test ears (n=7/group)

group	Weight of right ear after piercing (mg)	Weight of the left ear after piercing (mg)	swelling (mg)	Swelling inhibition rate (%)
control	21.71 (3.861)	13.86 (3.805)	7.90 (5.30)	0
CSS	18.71 (5.589)	12.29 (1.380)	6.40 (6.00)	18.99%
excipient PBO	19.71 (5.122)	12.00 (1.155)	7.70 (5.60)	2.53%
2.5% CSS	18.00 (2.082)	12.29 (1.799)	5.70 (3.10)	27.85%
5% CSS	17.71 (2.628)	13.14 (2.193)	3.90 (2.90)	50.63%
10% CSS	21.86 (4.981)	12.29 (2.430)	9.60 (4.70)	-21.52%
ASPL	14.00 (3.109)	10.71 (3.147)	3.30 (3.80)	58.23%

Note: There was no significant difference in swelling between the groups (P=0.177).

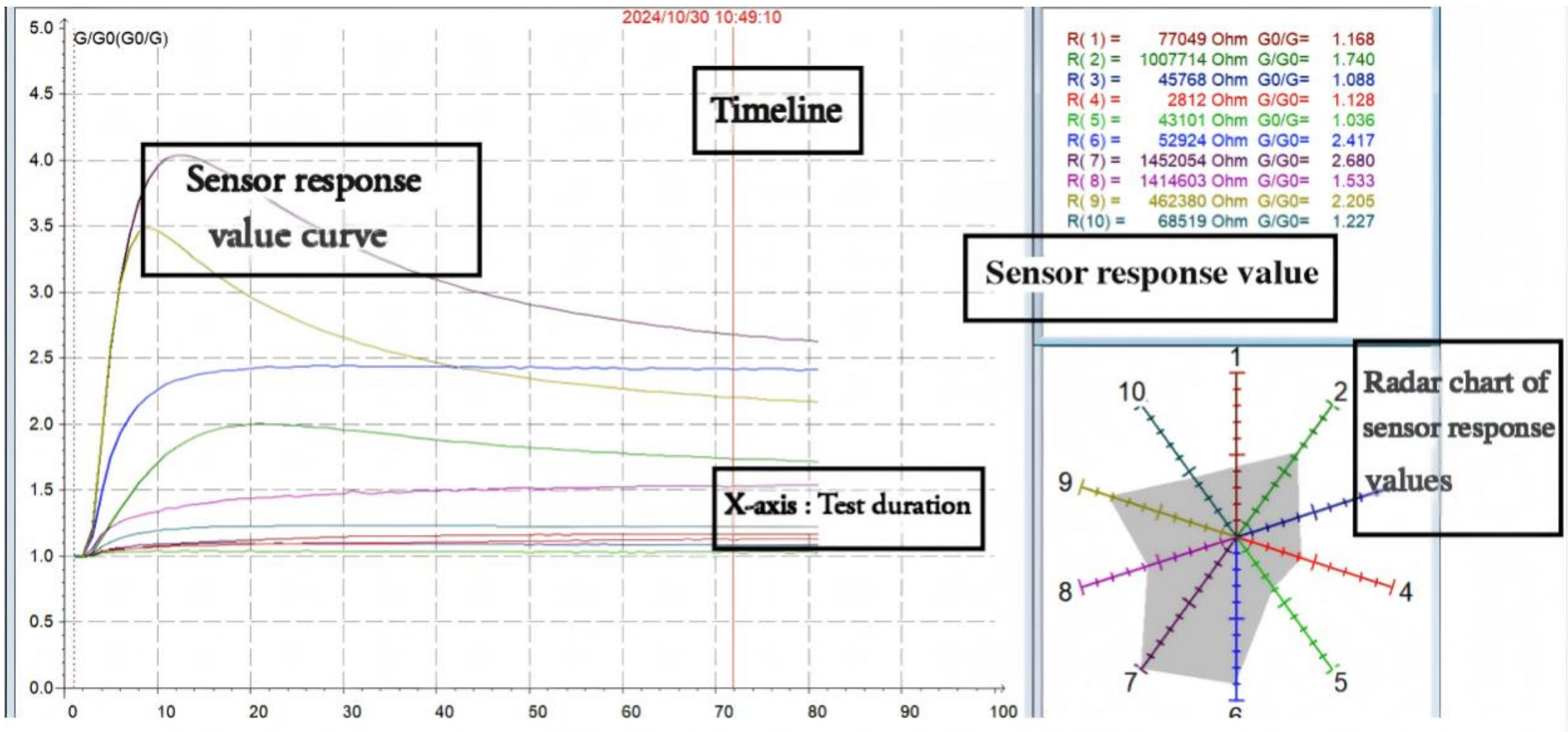

（CSS electronic nose data）

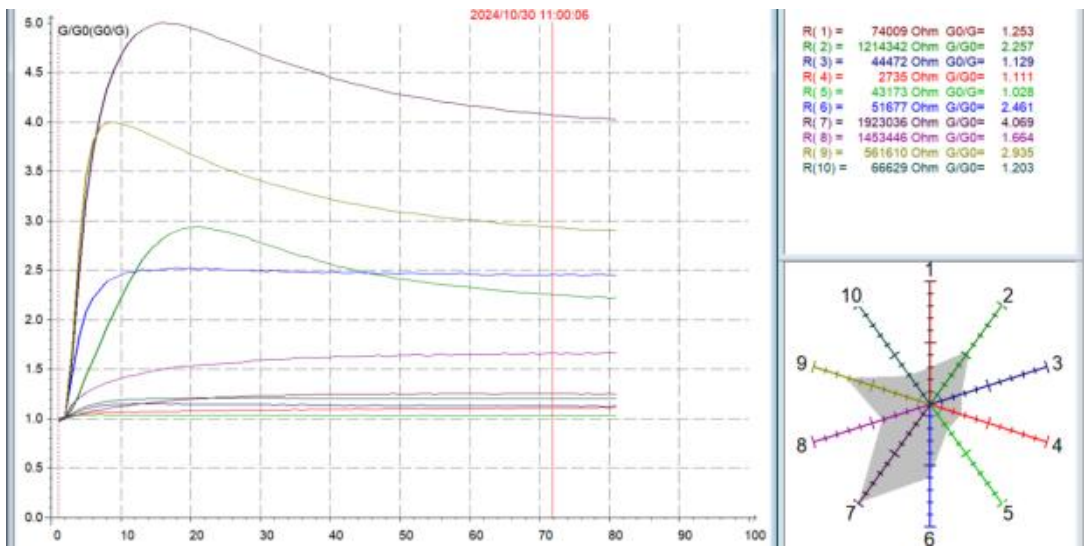

(excipient PBO)

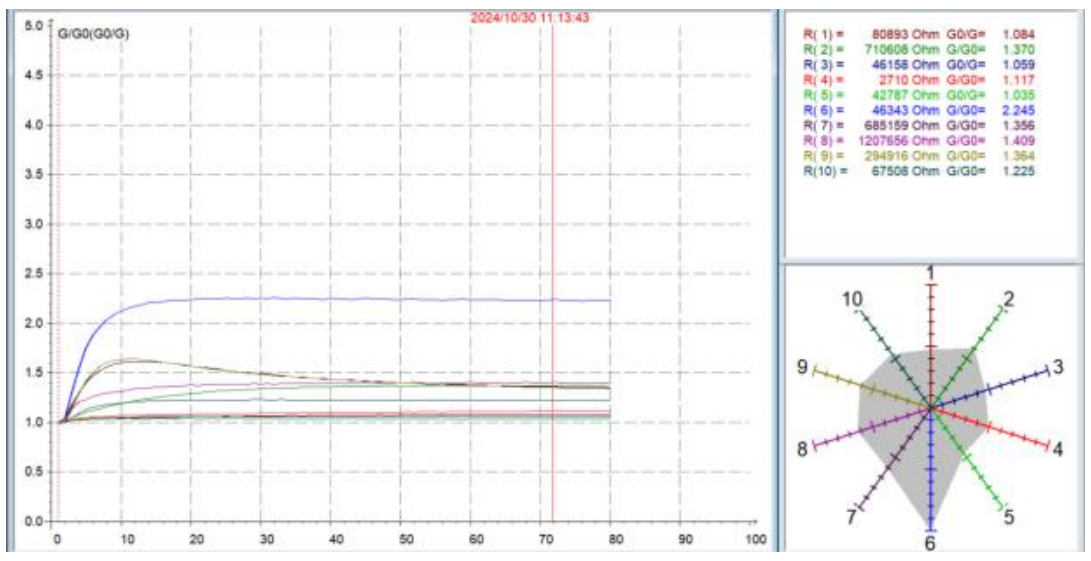

(2.5% CSS)

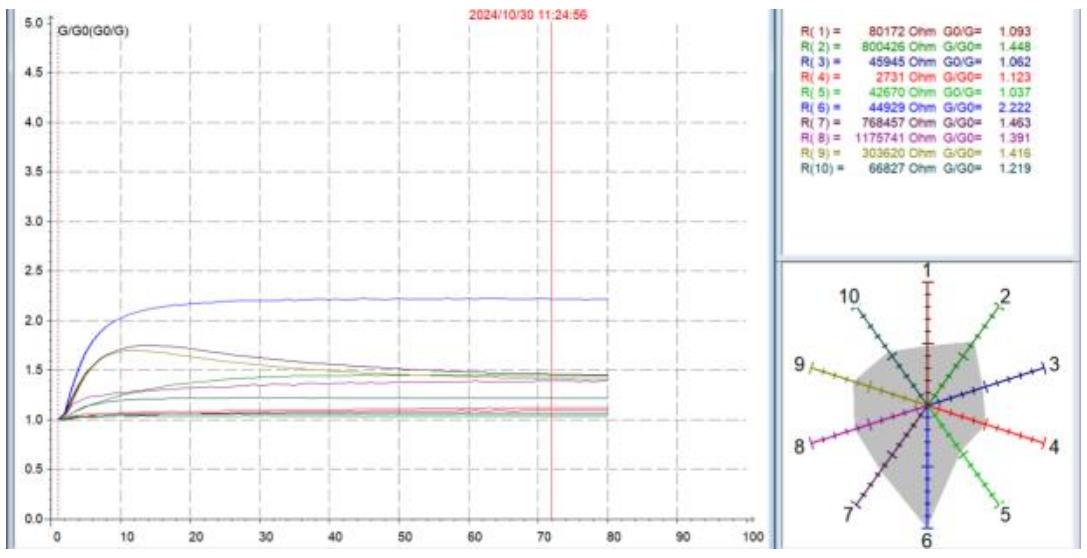

(5% CSS)

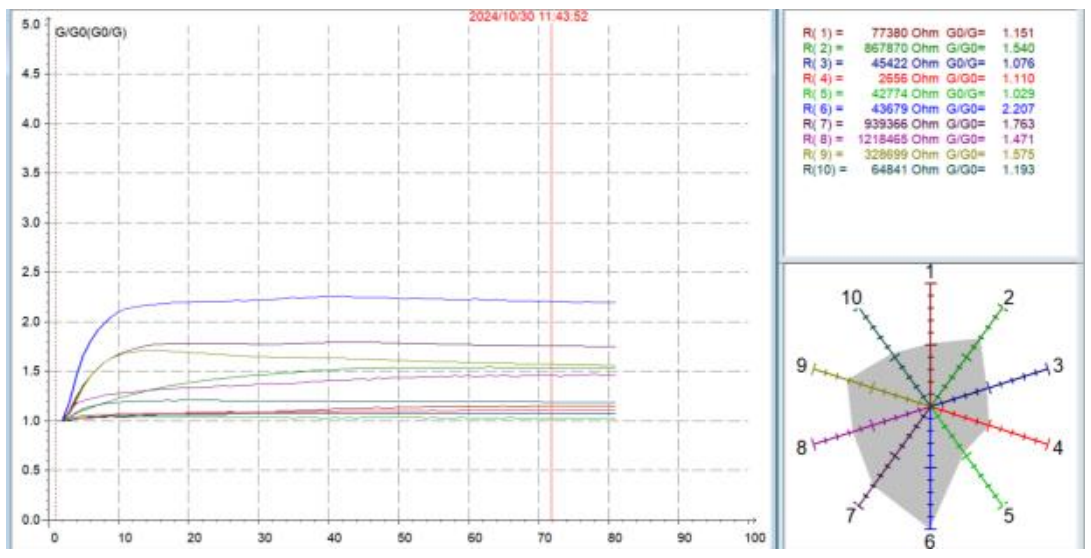

(10% CSS)

Figure S1 Electronic nose resistance response data of CSS and CSS simulated decoctions

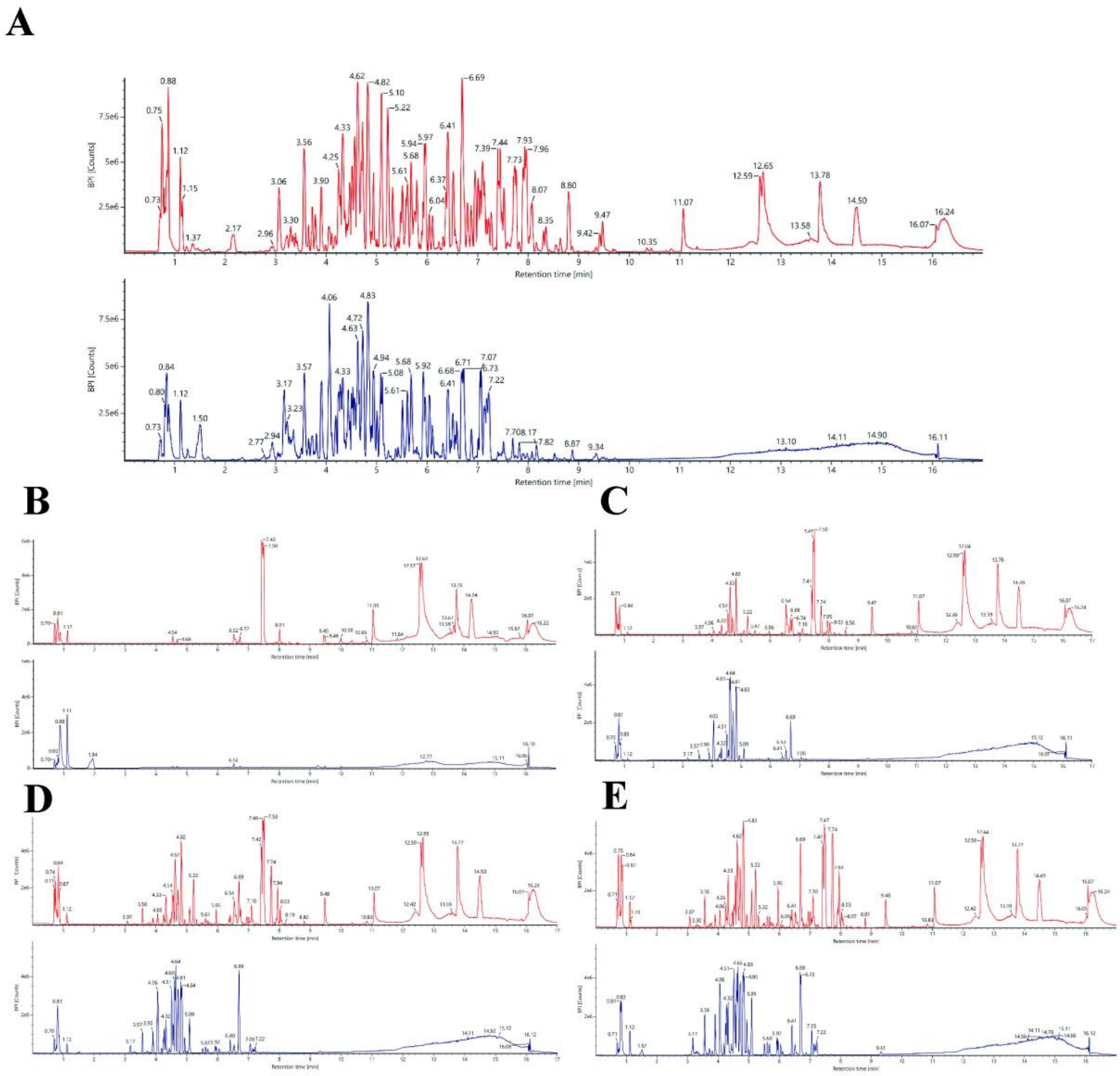

Figure S2 Analysis of relative concentrations of CSS and four placebo samples. Representative TICs obtained via UPLC-Q-TOF-MSE in positive (+) and negative (−) ion modes; red represents positive ions, and blue represents negative ions. A) CSS decoction extract (±); B) Excipient PBO TIC (±); C) 2.5% CSS TIC (±); D) TIC of 5% CSS (±); E) TIC of 10% CSS (±).

河北百草康神药业有限公司
产品检验报告

报告编号：**C24062930**

品　　名：	北柴胡	规　　格：	1kg×10 袋
产　　地：	河北承德	批　　号：	2406011
数　　量：	3475.3kg	样品来源：	包装工序
生产日期：	2024.06.11	样品编号：	JD202406080
请验日期：	2024.06.29	报告日期：	2024.06.29
检验依据：	《中国药典》2020 年版一部、四部		

检验项目	标准规定	检验结果
〖性状〗	本品呈不规则厚片。外表皮黑褐色或浅棕色，具纵皱纹和支根痕。切面淡黄白色，纤维性。质硬。气微香，味微苦。	本品呈不规则厚片。外表皮黑褐色，具纵皱纹和支根痕。切面淡黄白色，纤维性。质硬。气微香，味微苦。
〖鉴别〗	供试品色谱中，应在与对照药材色谱和对照品色谱相应的位置上，显相同颜色的斑点和荧光斑点	供试品色谱中，在与对照药材色谱和对照品色谱相应的位置上，显相同颜色的斑点和荧光斑点
〖检查〗	水分不得过 10.0%	6.1%
	总灰分不得过 8.0%	4.9%
	酸不溶性灰分不得过 3.0%	1.2%
	二氧化硫残留量不得过 150mg/kg	0mg/kg
〖浸出物〗	不得少于 11.0%	18.0%
〖含量测定〗	含柴胡皂苷 a 和柴胡皂苷 d 的总量不得少于 0.30%	0.54%

*引用北柴胡 240601 检验结果

检验结论：本品按《中国药典》2020 年版一部、四部检验，结果符合规定

报告人：韩旭娇　　审核人：李坤云　　批准人：[signature]

河北百草康神药业有限公司 质检专用章

河北百草康神药业有限公司
产品检验报告

报告编号：C24062444

**品　　名：**	陈皮	**规　　格：**	1kg×10 袋
**产　　地：**	四川盐边	**批　　号：**	2404022
**数　　量：**	2060.0kg	**样品来源：**	包装工序
**生产日期：**	2024.04.26	**样品编号：**	——
**请验日期：**	2024.06.24	**报告日期：**	2024.06.24
**检验依据：**	《中国药典》2020 年版一部、四部		

检验项目	标准规定	检验结果
〖性状〗	本品呈不规则的条状或丝状。外表面橙红色或红棕色，有细皱纹和凹下的点状油室；内表面浅黄白色，粗糙，附黄白色或黄棕色筋络状维管束。质稍硬而脆。气香，味辛、苦。	本品呈不规则的丝状。外表面橙红色，有细皱纹和凹下的点状油室；内表面浅黄白色，粗糙，附黄白色筋络状维管束。质稍硬而脆。气香，味辛、苦。
〖鉴别〗	（1）应具陈皮显微特征	具陈皮显微特征
	（2）供试品色谱中，应在与对照品色谱相应的位置上，显相同颜色的荧光斑点	供试品色谱中，在与对照品色谱相应的位置上，显相同颜色的荧光斑点
〖检查〗	水分不得过 13.0%	10.8%
	黄曲霉毒素　本品每 1000g 含黄曲霉毒素 B_1 不得过 5μg	未检出（检测限 0.004417μg/kg）
	含黄曲霉毒素 G_2、黄曲霉毒素 G_1、黄曲霉毒素 B_2 和黄曲霉毒素 B_1 的总量不得过 10μg	未检出（检测限 0.01666μg/kg）
	二氧化硫残留量不得过 150mg/kg	0mg/kg
〖含量测定〗	含橙皮苷不得少于 2.5%	5.0%

*引用陈皮 240402 检验结果

检验结论：本品按《中国药典》2020 年版一部、四部检验，结果符合规定

报告人：韩旭娅　　审核人：李坤云　　批准人：吕端端

河北百草康神药业有限公司 质检专用章

河北百草康神药业有限公司
产品检验报告

报告编号：C24062447

品 名：	川芎	规 格：	1kg×15 袋
产 地：	四川彭州	批 号：	2405012
数 量：	2595.0kg	样品来源：	包装工序
生产日期：	2024.05.13	样品编号：	JD202405055
请验日期：	2024.06.24	报告日期：	2024.06.24
检验依据：	《中国药典》2020 年版一部、四部		

检验项目	标准规定	检验结果
〖性状〗	本品为不规则厚片，外表皮灰褐色或褐色，有皱缩纹。切面黄白色或灰黄色，具有明显波状环纹或多角形纹理，散生黄棕色油点。质坚实。气浓香，味苦、辛，微甜。	本品为不规则厚片，外表皮灰褐色，有皱缩纹。切面灰黄色，具有明显波状环纹，散生黄棕色油点。质坚实。气浓香，味苦、辛，微甜。
〖鉴别〗	（1）应具川芎显微特征	具川芎显微特征
	（2）应呈正反应	呈正反应
	（3）供试品色谱中，应在与对照药材色谱和对照品色谱相应的位置上，显相同颜色的斑点	供试品色谱中，在与对照药材色谱和对照品色谱相应的位置上，显相同颜色的斑点
〖检查〗	水分不得过 12.0%	8.6%
	总灰分不得过 6.0%	4.0%
	二氧化硫残留量不得过 150mg/kg	0mg/kg
〖浸出物〗	不得少于 12.0%	37.9%
〖含量测定〗	含阿魏酸不得少于 0.10%	0.21%

*引用川芎 240501 检验结果

检验结论：本品按《中国药典》2020 年版一部、四部检验，结果符合规定

报告人：韩旭娇　　审核人：李坤云　　批准人：吕瑞瑞

（印章：河北百草康神药业有限公司 质检专用章）

河北百草康神药业有限公司
产品检验报告

报告编号：C24063028

**品　　名：**	醋香附	**规　　格：**	1kg×15 袋
**产　　地：**	广西梧州	**批　　号：**	2406011
**数　　量：**	1828.5kg	**样品来源：**	包装工序
**生产日期：**	2024.06.13	**样品编号：**	JD202406078
**请验日期：**	2024.06.29	**报告日期：**	2024.06.30
**检验依据：**	《中国药典》2020 年版一部、四部		

检验项目	标准规定	检验结果
〖性状〗	本品形如香附片（粒），表面黑褐色。微有醋香气，味微苦。	本品形如香附片（粒），表面黑褐色。微有醋香气，味微苦。
〖鉴别〗	（1）应具醋香附显微特征	具醋香附显微特征
	（2）供试品色谱中，应在与对照品色谱相应的位置上，显相同的深蓝色斑点；喷以二硝基苯肼试液，放置片刻，斑点渐变为橙红色	供试品色谱中，在与对照品色谱相应的位置上，显相同的深蓝色斑点；喷以二硝基苯肼试液，放置片刻，斑点渐变为橙红色
〖检查〗	水分不得过 13.0%	7.9%
	总灰分不得过 4.0%	2.0%
	二氧化硫残留量不得过 150mg/kg	0mg/kg
〖浸出物〗	不得少于 13.0%	19.2%
〖含量测定〗	含挥发油不得少于 0.8%	1.0%

*引用醋香附 240601 检验结果

检验结论：本品按《中国药典》2020 年版一部、四部检验，结果符合规定

报告人：韩旭娥　　审核人：李坤云　　批准人：[signature]

河北百草康神药业有限公司 质检专用章

河北百草康神药业有限公司
产品检验报告

报告编号：**C24082571**

品　　名：	白芍	规　　格：	1kg×15 袋
产　　地：	安徽亳州	批　　号：	2408011
数　　量：	3410.8kg	样品来源：	包装工序
生产日期：	2024.08.20	样品编号：	——
请验日期：	2024.08.25	报告日期：	2024.08.25
检验依据：	《中国药典》2020 年版、《国家中药饮片炮制规范》		

检验项目	标准规定	检验结果
〖性状〗	本品呈类圆形的薄片。表面淡棕红色或类白色。切面类白色或微带棕红色，形成层环明显，可见稍隆起的筋脉纹呈放射状排列。气微，味微苦、酸。	本品呈类圆形的薄片。表面淡棕红色，切面类白色，形成层环明显，可见稍隆起的筋脉纹呈放射状排列。气微，味微苦、酸。
〖鉴别〗	（1）应具白芍显微特征	具白芍显微特征
	（2）供试品色谱中，应在与对照品色谱相应的位置上，显相同的蓝紫色斑点	供试品色谱中，在与对照品色谱相应的位置上，显相同的蓝紫色斑点
〖检查〗	水分不得过 14.0%	10.3%
	总灰分不得过 4.0%	2.9%
	二氧化硫残留量不得过 400mg/kg	122mg/kg
〖浸出物〗	不得少于 22.0%	28.5%
〖含量测定〗	含芍药苷不得少于 1.2%	2.6%

*引用白芍 240801 检验结果

检验结论：本品按《中国药典》2020 年版、《国家中药饮片炮制规范》检验，结果符合规定

报告人：韩旭娇　　审核人：李坤云　　批准人：吕瑞瑞

河北百草康神药业有限公司 质检专用章

河北百草康神药业有限公司
产品检验报告

报告编号：C24062156

品　　名：	麸炒枳壳	规　　格：	1kg×15 袋
产　　地：	江西吉安	批　　号：	2406011
数　　量：	1792.4kg	样品来源：	包装工序
生产日期：	2024.06.07	样品编号：	JD202406048
请验日期：	2024.06.21	报告日期：	2024.06.21
检验依据：	《中国药典》2020 年版一部、四部		

检验项目	标准规定	检验结果
〖性状〗	本品形如枳壳片，色较深，偶有焦斑。	本品形如枳壳片，色较深，偶有焦斑。
〖鉴别〗	（1）应具麸炒枳壳显微特征	具麸炒枳壳显微特征
	（2）供试品色谱中，应在与对照品色谱相应的位置上，呈相同颜色的荧光斑点	供试品色谱中，在与对照品色谱相应的位置上，呈相同颜色的荧光斑点
〖检查〗	水分不得过 12.0%	5.8%
	总灰分不得过 7.0%	4.2%
	二氧化硫残留量不得过 150mg/kg	0mg/kg
〖含量测定〗	含柚皮苷不得少于 4.0%	5.7%
	含新橙皮苷不得少于 3.0%	4.6%

*引用麸炒枳壳 240601 检验结果

检验结论：本品按《中国药典》2020 年版一部、四部检验，结果符合规定

报告人：韩旭娘　　审核人：李坤玉　　批准人：占璐璐

河北百草康神药业有限公司 质检专用章

河北百草康神药业有限公司
产品检验报告

报告编号：C24070939

品　名：	甘草片	规　格：	1kg×15 袋
产　地：	新疆伊犁	批　号：	2406011
数　量：	3369.4kg	样品来源：	包装工序
生产日期：	2024.06.11	样品编号：	JD202406044
请验日期：	2024.07.09	报告日期：	2024.07.09
检验依据：	《中国药典》2020 年版一部、四部		

检验项目	标准规定	检验结果
〖性状〗	本品呈类圆形的厚片。外表皮红棕色或灰棕色，具纵皱纹。切面略显纤维性，中心黄白色，有明显放射状纹理及形成层环。质坚实，具粉性。气微，味甜而特殊。	本品呈类圆形的厚片。外表皮红棕色，具纵皱纹。切面略显纤维性，中心黄白色，有明显放射状纹理及形成层环。质坚实，具粉性。气微，味甜而特殊。
〖鉴别〗	（1）应具甘草片显微特征	具甘草片显微特征
	（2）供试品色谱中，应在与对照药材色谱相应的位置上，显相同颜色的荧光斑点；应在与对照品色谱相应的位置上，显相同的橙黄色荧光斑点	供试品色谱中，在与对照药材色谱相应的位置上，显相同颜色的荧光斑点；在与对照品色谱相应的位置上，显相同的橙黄色荧光斑点
〖检查〗	水分不得过 12.0%	5.2%
	总灰分不得过 5.0%	3.2%
	重金属及有害元素	
	铅不得过 5mg/kg	0.1mg/kg
	镉不得过 1mg/kg	0mg/kg
	砷不得过 2mg/kg	0.1mg/kg
	汞不得过 0.2mg/kg	0.03mg/kg
	铜不得过 20mg/kg	4.8mg/kg
	二氧化硫残留量不得过 150mg/kg	0mg/kg
〖含量测定〗	含甘草苷不得少于 0.45%	0.69%
	含甘草酸不得少于 1.8%	2.0%

*引用甘草片 240601 检验结果

检验结论：本品按《中国药典》2020 年版一部、四部检验，结果符合规定

河北百草康神药业有限公司 质检专用章

报告人：韩旭娥　　审核人：李坤云　　批准人：吕谛谛
