# Supplementary material for: Multidimensional evaluation of four chaihu-shugan-san simulated decoction for use as placebo controls: a comparative study
Source: Front Pharmacol. 2026 Aug 4;17:1806757. doi: 10.3389/fphar.2026.1806757 (PMC13481859; doi:10.3389/fphar.2026.1806757)
Supplement: Supplementary file 2 [file DataSheet2.pdf]

Supplementary Material 3: Chemical Characterization of CSS and CSS Simulated Decoction

（Preparation of Type B alternatives in accordance with the ConPhyMP expert consensus）

1 Botanical drug Information and Extraction Methods

1.1 Composition of the Compound and Identification of Constituents

The composition of CSS comprises seven botanical drugs, whose botanical names and complete species information have been verified through the Pharmacopoeia of the People's Republic of China and the http://mpns.kew.org (MPNS) database, as detailed in Table 1.The botanical sources of each botanical drug ingredient were identified in accordance with the *Pharmacopoeia of the People's Republic of China* (2020 Edition). The composition and proportions of the compound are shown in Table S1.

表 S1 Composition of the Compound (CSS)

	Latin Name (including author)	Chinese Name	Family	Part used	Weight, g	Pharmacopoeia Reference
1	*Bupleurum falcatum* L.	Chai Hu	Apiaceae	Root	12	Chinese Pharmacopoeia
2	*Citrus reticulata Blanco*	Chen Pi	Rutaceae	Rind	12	Chinese Pharmacopoeia
3	*Conioselinum anthriscoides 'Chuanxiong'*	Chuan Xiong	Apiaceae	Root and rhizoma	12	Chinese Pharmacopoeia
4	*Cyperus rotundus* L.	Xiang Fu	Cyperaceae	Root and rhizoma	9	Chinese Pharmacopoeia
5	*Paeonia lactiflora Pall.*	Shao Yao	Paeoniaceae	Root	15	Chinese Pharmacopoeia
6	*Citrus* × *aurantium* L. aurantium	Zhi Qiao	Rutaceae	Fruit	12	Chinese Pharmacopoeia
7	*Glycyrrhiza glabra* L	Gan Cao	Fabaceae	Root and rhizoma	6	Chinese Pharmacopoeia

1.2 Preparation of Test Samples

1.2.1 Preparation of Test Samples for HRMS

Methanol extract: Accurately measure out 0.2 mL decoction, add three times the volume of purified water, and sonicate for 20 minutes. Centrifuge at 12,000 × g for 10 minutes, collect the supernatant, filter through a 0.22 μm microporous membrane, and store at 4 °C for later use.

Ethyl acetate extraction fraction: Take 0.2 mL decoction of the methanol extract described above, add an equal volume of ethyl acetate, shake to extract, centrifuge at 12,000 × g for 10 min, collect the ethyl acetate layer, dry under nitrogen, redissolve in 0.4 mL of methanol, centrifuge at 12,000 × g for 10 min, then collect the supernatant and store at 4 °C for later use.

Preparation of mixed standard solution: Take appropriate amounts of paeoniflorin, glycyrrhizin, naringin, ligustrin I, ligustrin H, glycyrrhizin, glycyrrhetic acid, bupleurin B1, and bupleurin D in appropriate amounts, and prepare a 2 mg/mL stock solution in methanol. Mix the stock solutions of each standard in equal proportions, then dilute with methanol to prepare a mixed standard solution with a concentration of 50 μg/mL for each standard, and set aside for later use.

1.2.2 Preparation of Test Samples for MRM Quantification

Accurately weigh appropriate amounts of glycyrrhizin, bupleuroside D, and paeonol glycoside reference standards, and prepare a 2 mg/mL stock solution using methanol. Take the stock solutions of each standard and dilute them with methanol to prepare a series of mixed standard working solutions (50–5000 ng/mL) for injection and analysis.

Accurately measure out 0.2 mL decoction, add 6 mL of methanol, and sonicate for 20 min. Centrifuge at 12,000 × g for 10 min, take the supernatant, dilute it 10-fold and 50-fold with methanol, filter through a 0.22 μm microporous membrane, and store at 4 °C for later use. Select the dilution results within the linear range (50–5000 ng/mL) and multiply them by the corresponding dilution factor to calculate the sample content.

1.3 Reference Standard Information

Information on the reference standards used in this study is provided in Table S2.

Table S2 Reference Standard Information

	English Name	Molecular Formula	CAS Number	Purity	Supplier	Lot Number	Application
1	Albiflorin	C23H28O11	39011-90-0	≥98%	Chem Faces	CFS202102	HRMS + MRM
2	Liquiritin	C21H22O9	551-15-5	≥98%	源叶生物	P25J7F18328	HRMS + MRM
3	Hesperidin	C28H34O15	520-26-3	≥98%	源叶生物	K02M3C1	仅 HRMS
4	Senkyunolide I	C12H16O4	94596-28-8	≥98%	Chem Faces	CFS202102	仅 HRMS
5	Senkyunolide H	C12H16O4	94596-27-7	≥98%	瑞芬思德	RFS-	仅 HRMS

					丹	Y08402112008	
6	Liquiritigenin	$C_{15}H_{12}O_4$	578-86-9	≥98%	源叶生物	ZM0314BD14	仅 HRMS
7	Glycyrrhizic acid	$C_{42}H_{62}O_{16}$	1405-86-3	≥98%	源叶生物	P04N7F24311	仅 HRMS
8	Saikosaponin B1	$C_{42}H_{68}O_{13}$	58558-08-0	≥98%	瑞芬思德丹	RFS-C02602005022	仅 HRMS
9	Saikosaponin D	$C_{42}H_{68}O_{13}$	20874-52-6	≥98%	瑞芬思德丹	RFS-C08502111026	HRMS + MRM

2 High-Resolution Liquid Chromatography-Mass Spectrometry (LC-HRMS) Chemical Fingerprint Profiles

2.1 Instruments and Materials

A Waters Acquity UPLC ultra-high-performance liquid chromatography system was coupled with a Synapt G2-Si HDMS high-resolution ion mobility mass spectrometer (Waters Corporation, USA). The column used was an Acquity UPLC BEH C18 (2.1 mm × 100 mm, 1.7 µm, Waters); column temperature was 35 °C; and the sample compartment temperature was 4 °C. Reagents: methanol (chromatographic grade), acetonitrile (chromatographic grade), and formic acid (chromatographic grade) were all purchased from Fisher.

2.2 LC and MS Conditions

LC conditions: Mobile phase A was a 0.1% formic acid aqueous solution; mobile phase B was a 0.1% formic acid acetonitrile solution; flow rate 0.3 mL/min; injection volume 2.0 µL. 30-minute gradient elution conditions: 0–0.8 min, 95% A; 0.8–5.0 min, 95%–88% A; 5.0–9.0 min, 88%–80% A; 9.0–11.0 min, 80%–75% A; 11.0–18.0 min, 75%–55% A; 18.0–25.0 min, 55%–1% A; 25.0–28.0 min, 1% A; 28.1–30.0 min, 95% A.

MS conditions: Mass spectrometry data were acquired in both positive and negative ion electrospray ionization (ESI) modes. Capillary voltage: 3.5 kV for positive ion mode, 2.2 kV for negative ion mode; Desolvation gas temperature: 450 °C for positive ion mode, 400 °C for negative ion mode; Ion source temperature: 110 °C; Desolvation gas flow rate: 800 L/h; Scan range: m/z 50–1200; Collision energy (CE): 30–60 V. The mass axis was calibrated using a sodium formate solution. Data acquisition software: MassLynx V4.1 (Waters).

2.3 Identification Results

Characteristic components were identified by comparing the retention times and exact molecular masses of the reference standards with those of the samples. A total of 9 characteristic peaks were identified, all of which were verified against standard samples. The results are shown in Table S3, and the chromatograms are shown in Figures S1–S4.

Table S3 Information on signature compounds identified by LC-HRMS (CSS, 30-minute gradient)

	English Name	Exact Molecular Weight	Mass (m/z)	Molecular Formula	Retention Time (min)	Sum Ions	Drug Source
1	Albiflorin	480.16316	525.16230	C23H28O11	6.77	[M+FA-H]-	*Paeonia lactiflora Pall.*
2	Liquiritin	418.12638	417.11858	C21H22O9	8.65	[M-H]-	*Glycyrrhiza glabra* L
3	Hesperidin	610.18977	609.18200	C28H34O15	10.71	[M-H]-	*Citrus reticulata Blanco*、*Citrus* × *aurantium* L. aurantium
4	Senkyunolide I	224.10486	247.09490	C12H16O4	11.34	[M+Na]+	Conioselinum anthriscoides 'Chuanxiong'
5	Senkyunolide H	224.10486	247.09510	C12H16O4	11.92	[M+Na]+	Conioselinum anthriscoides 'Chuanxiong'
6	Liquiritigenin	256.07356	257.08140	C15H12O4	12.14	[M+H]+	*Glycyrrhiza glabra* L
7	Glycyrrhizic acid	822.40379	823.41030	C42H62O16	17.06	[M+H]+	*Glycyrrhiza glabra* L
8	Saikosaponin B1	780.46599	781.47260	C42H68O13	19.19	[M+H]+	*Bupleurum falcatum* L.
9	Saikosaponin D	780.46599	781.47260	C42H68O13	20.31	[M+H]+	*Bupleurum falcatum* L.

2.4 Fingerprint Chromatograms of the Methanol Extract

The base peak chromatograms (BPCs) of the methanol extract and the mixed reference standards in both positive and negative ion modes are shown in Figures S1–S2. The numerical labels in the figures correspond to the compound numbers in Table S3, and the vertical dashed lines are anchored at the peak tops of the reference standards.

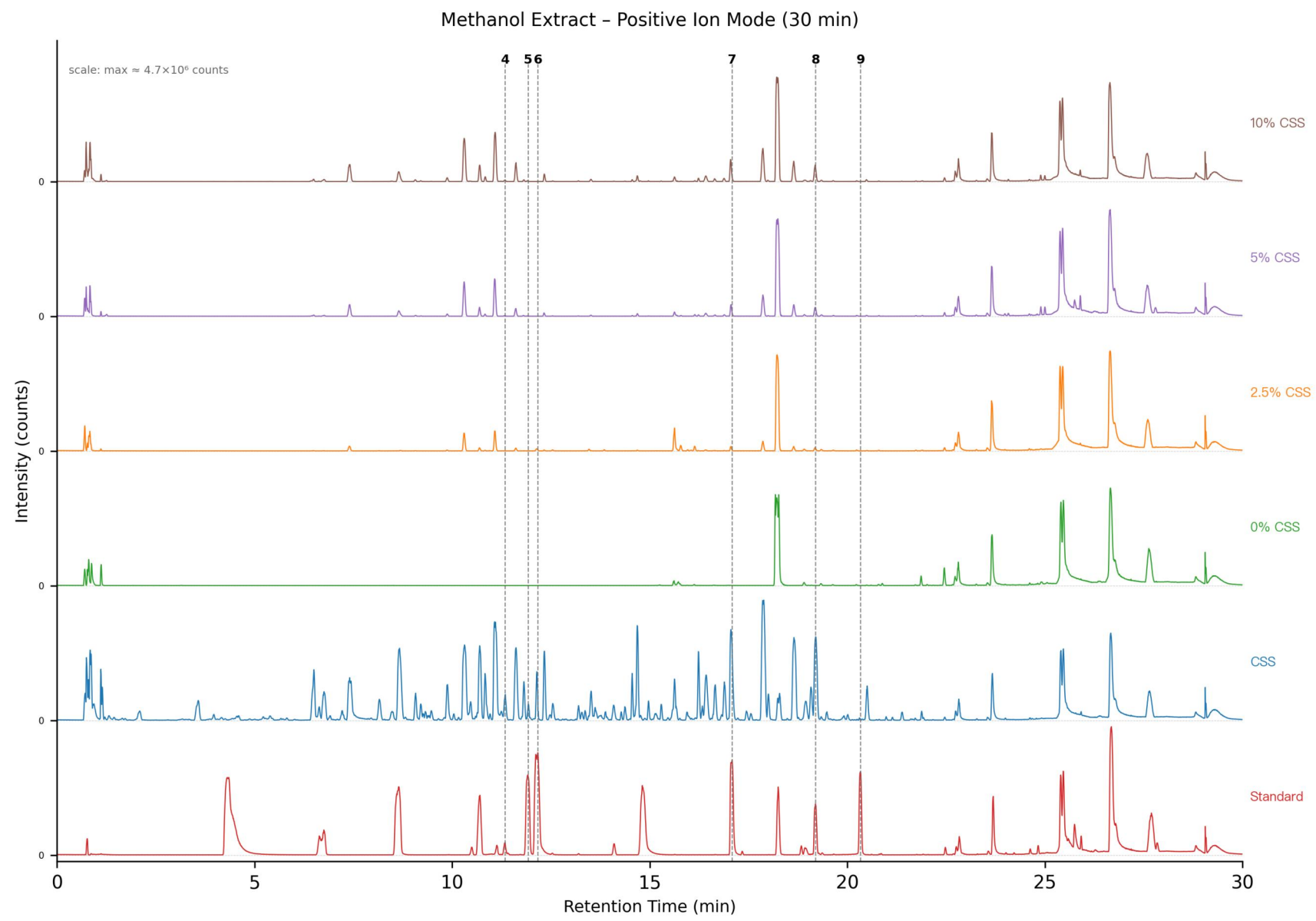

Figure S1 BPC chromatogram of the methanol extract (30 min, positive ion mode). From bottom to top: standard, CSS, 0% CSS, 2.5% CSS, 5% CSS, 10% CSS; see Table S3 for numerical labels.

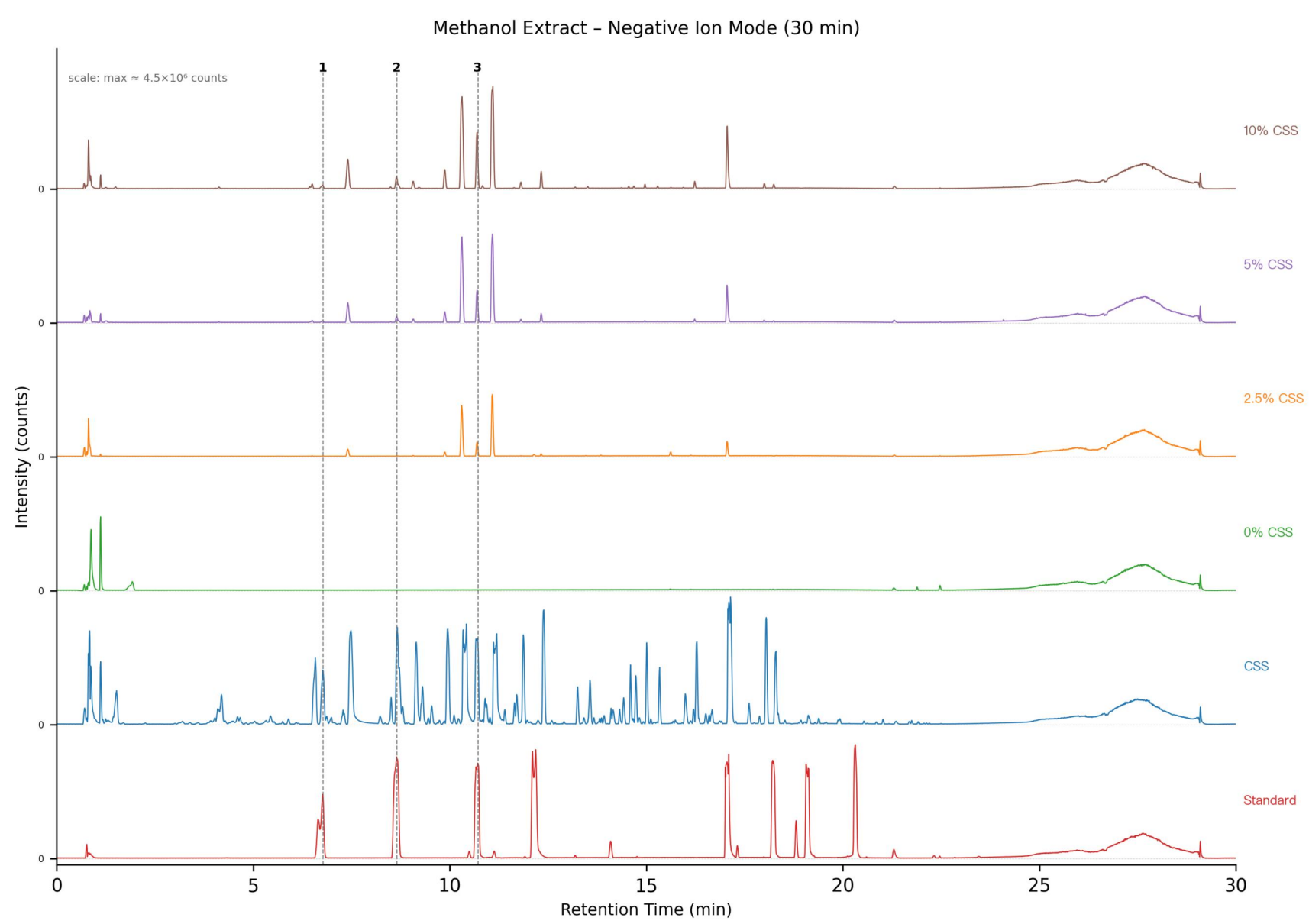

Figure S2 BPC chromatogram of the methanol extract (30 min, negative ion mode). From bottom to top: standard, CSS, 0% CSS, 2.5% CSS, 5% CSS, 10% CSS; see Table S3 for numerical labels.

2.5 Fingerprint Chromatograms of Ethyl Acetate-Extracted Fractions

The chromatograms of the ethyl acetate-extracted fractions and the mixed reference standards in both positive and negative ion modes are shown in Figures S3–S4.

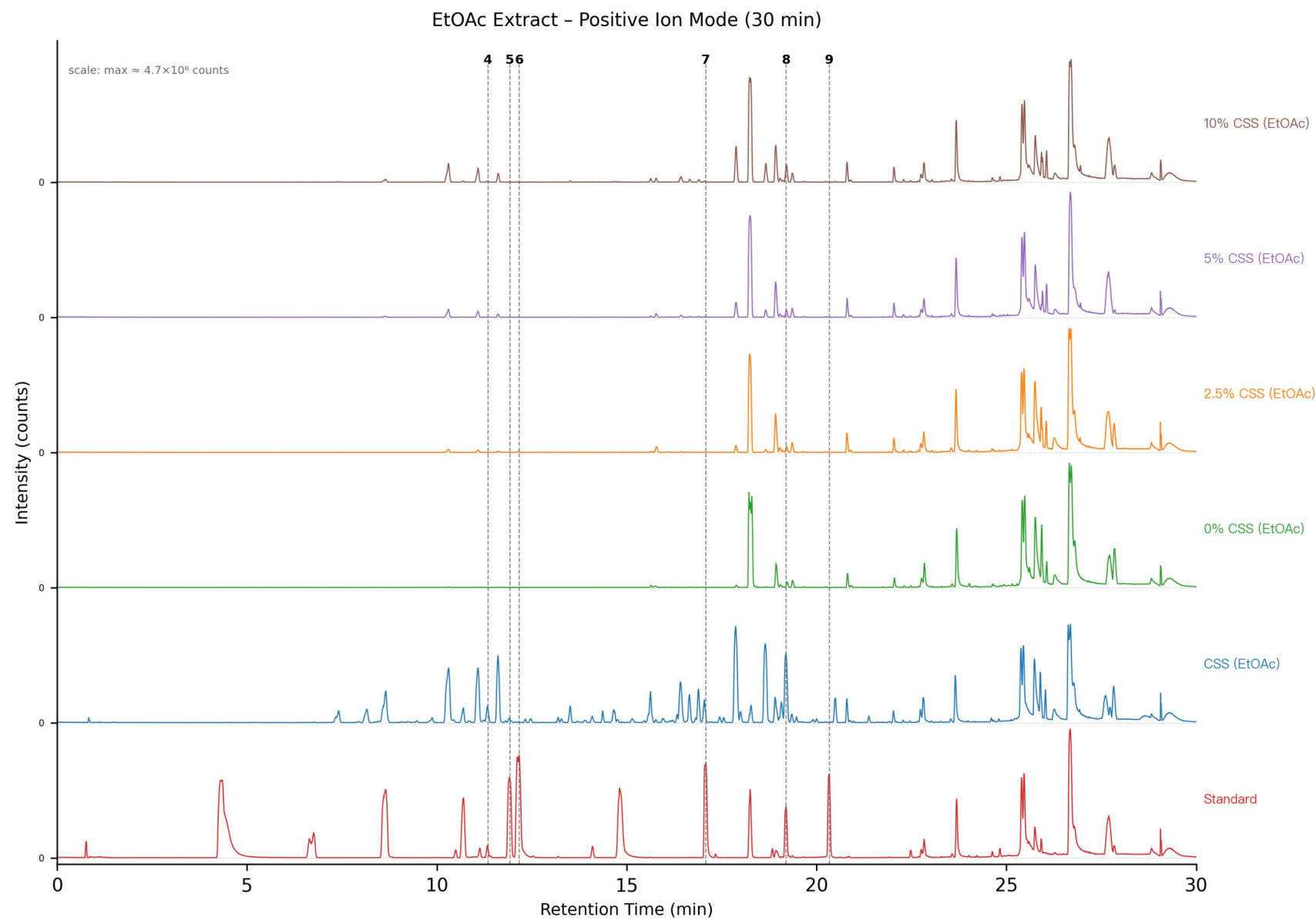

Figure S3 BPC of the ethyl acetate-extracted fraction B (30 min, positive ion mode). From bottom to top: standard, CSS (EtOAc), 0%–10% CSS (EtOAc); see Table S3 for numerical labels.

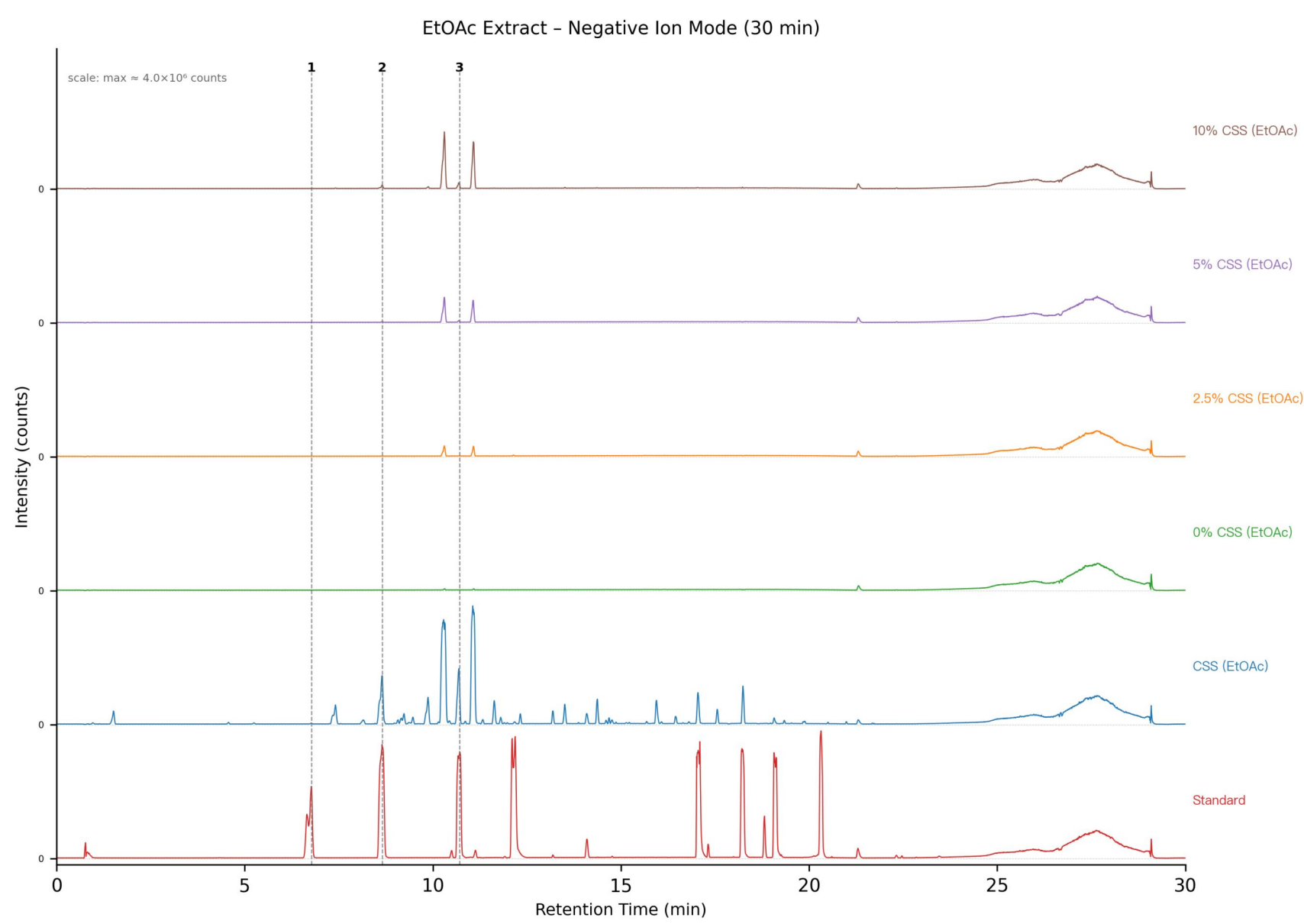

Figure S4 BPC chromatogram of the ethyl acetate extract from region B (30 min, negative ion mode). From bottom to top: standard, CSS (EtOAc), 0%–10% CSS (EtOAc); see Table S3 for numerical labels.

3 Triple Quadrupole MRM Targeted Quantification

3.1 Instrumentation and Conditions

Quantitative analysis was performed using an [AB SCIEX 5500] triple quadrupole mass spectrometer coupled with a Waters Acquity UPLC ultra-high-performance liquid chromatography system (Waters Corporation, USA). Reagents: Methanol (chromatographic grade), acetonitrile (chromatographic grade), and formic acid (chromatographic grade) were all purchased from Fisher.

3.2 LC and MS Conditions

LC Conditions: Column: Kinetex 2.6 μm EVO C18 (Phenomenex, 2.1 mm × 100 mm); Mobile phase A: 0.1% formic acid in water; Mobile phase B: acetonitrile; Flow rate: 0.3 mL/min; Injection volume: 2.0 μL; Column temperature: 40 °C; Sample compartment temperature: 4 °C. Gradient elution conditions (8 min): 0–0.5 min, 95% A; 0.5–5.0 min, 95%–1% A; 5.0–6.5 min, 1% A; 6.5–6.7 min, 1%–95% A; 6.7–8.0 min, 95% A.

MS conditions: Electrospray ionization (ESI), multiple reaction monitoring (MRM) mode, with simultaneous scanning of positive and negative ions. The MRM ion pairs and optimized parameters for the three quantified components are shown in Table S4.

Table S4 MRM ion pairs and mass spectrometry optimization parameters

	Compound	Precursor Ion (m/z)	Product Ion (m/z)	DP (V)	CE (V)	Ion Mode
1	Liquiritin	417.2	255.0	−160	−17	Negative ions
2	Saikosaponin D	779.6	617.4	−160	−46	Negative ions
3	Albiflorin	481.4	197.2	+150	+13	Negative ions

3.3 Method Validation

3.3.1 Linearity

A series of mixed standard solutions was prepared (7 concentration levels: 50, 250, 500, 1000, 2500, 5000 ng/mL), and a weighted linear regression (weight $1/x^2$) was performed using peak area (y) against concentration (x, ng/mL). The regression equations and correlation coefficients for each batch within the linear range are shown in Table S5.

Table S5 Linear relationships for the three quantitative components

	Compound	Batch	Linear Range(ng/mL)	Regression Equation	r	Weighted
1	Liquiritin	Batch 1	50–5000 ng/mL	$y = 1210x + 3650$	0.9998	$1/x^2$
		Batch 2	50–5000 ng/mL	$y = 1100x + 3270$	0.9993	$1/x^2$
		Batch 3	50–5000 ng/mL	$y = 1020x + 1460$	0.9996	$1/x^2$

2	Saikosaponin D	Batch 1	50–5000 ng/mL	$y = 850x - 103$	0.9964	$1/x^2$
		Batch 2	50–5000 ng/mL	$y = 779x + 1110$	0.9989	$1/x^2$
		Batch 3	50–5000 ng/mL	$y = 718x + 1170$	0.9983	$1/x^2$
3	Albiflorin	Batch 1	50–5000 ng/mL	$y = 3100x + 8940$	0.9997	$1/x^2$
		Batch 2	50–5000 ng/mL	$y = 2710x + 543$	0.9997	$1/x^2$
		Batch 3	50–5000 ng/mL	$y = 2340x + 2960$	0.9997	$1/x^2$

3.3.2 Precision and Accuracy

Using a mixed standard solution, quality control samples were prepared at three concentration levels: low (LQC, 100 ng/mL), medium (MQC, 2000 ng/mL), and high (HQC, 3750 ng/mL), with three replicates per batch. Three consecutive batches were analyzed. Precision was evaluated using the relative standard deviation (RSD%) of peak area, while accuracy was expressed as the ratio of measured concentration to theoretical concentration (%). The results are shown in Table S6.

Table S6 Precision and Accuracy of Three Quantitative Components

	Compound	QC Level	Theoretical Concentration (ng/mL)	Batch	Measured Concentration（Mean±SD）(ng/mL)	Accuracy (%)	Precision RSD (%)
1	Liquiritin	LQC	100	Batch 1	96.6 ± 2.2	96.6	2.2
				Batch 2	92.7 ± 1.9	92.7	2.1
				Batch 3	97.4 ± 1.9	97.4	2.0
				Inter-batch	95.6 ± 2.5	95.6	2.6
		MQC	2000	Batch 1	1883.3 ± 66.6	94.2	3.5
				Batch 2	1840.0 ± 43.6	92.0	2.4
				Batch 3	1973.3 ± 28.9	98.7	1.5
				Inter-batch	1898.9 ± 68.0	95.0	3.6
		HQC	3750	Batch 1	3490.0 ± 36.1	93.1	1.0
				Batch 2	3396.7 ± 135.8	90.5	4.0
				Batch 3	3566.7 ± 40.4	95.1	1.1
				Inter-batch	3484.4 ± 85.1	92.9	2.4
2	Saikosaponin D	LQC	100	Batch 1	94.3 ± 1.5	94.3	1.6
				Batch 2	93.4 ± 2.8	93.4	3.0
				Batch 3	95.5 ± 2.0	95.5	2.1
				Inter-batch	94.4 ± 1.1	94.4	1.1
		MQC	2000	Batch 1	1906.7 ± 40.4	95.2	2.1
				Batch 2	1913.3 ± 47.3	95.5	2.5
				Batch 3	1970.0 ± 50.0	98.5	2.5
				Inter-batch	1930.0 ± 34.8	96.4	1.8
		HQC	3750	Batch 1	3376.7 ± 55.1	90.0	1.6
				Batch 2	3370.0 ± 65.6	89.8	1.9
				Batch 3	3433.3 ± 80.2	91.6	2.3
				Inter-batch	3393.3 ± 34.8	90.5	1.0
3	Albiflorin	LQC	100	Batch 1	98.9 ± 3.0	98.9	3.0
				Batch 2	96.8 ± 0.8	96.8	0.8
				Batch 3	96.9 ± 1.5	96.9	1.5
				Inter-batch	97.5 ± 1.2	97.5	1.2
		MQC	2000	Batch 1	1976.7 ± 125.0	98.9	6.3
				Batch 2	1933.3 ± 55.1	96.6	2.8
				Batch 3	2016.7 ± 37.9	100.9	1.9
				Inter-batch	1975.6 ± 41.7	98.8	2.1
		HQC	3750	Batch 1	3586.7 ± 113.7	95.7	3.2
				Batch 2	3546.7 ± 140.5	94.6	4.0
				Batch 3	3683.3 ± 85.0	98.4	2.3
				Inter-batch	3605.6 ± 70.3	96.2	1.9

3.4 Results of Quantitative Analysis

The concentrations of the three quantified components in the CSS and samples from the different treatment groups are shown in Table S7. ND indicates that the concentration in the dilution series was below the limit of quantification (< 50 ng/mL).

Table S7 Results of the determination of the concentrations of three quantified components in samples from each CSS processing group

	Compound	0% CSS	2.5% CSS	5% CSS	10% CSS	CSS	Selection Criteria
1	Liquiritin	ND	ND	6.42μg/mL	12.45μg/mL	110 μg/mL	Characteristic flavonoid glycosides of licorice; components subject to content determination in the Chinese Pharmacopoeia
2	Saikosaponin D	ND	ND	ND	ND	0.06 μg/mL	The primary triterpenoid saponins in Bupleurum exhibit significant anti-inflammatory pharmacological activity.
3	Albiflorin	ND	2.975μg/mL	6.99 μg/mL	13.85μg/mL	120.725 μg/mL	The main monoterpenoid glycosides in white peony root; a component subject to content determination in the Chinese Pharmacopoeia

3.5 MRM Chromatograms

The MRM chromatograms of the blank control, limit of quantification (LLOQ), and test solution for the three quantified compounds are shown in Figure S6(A)–(C). No significant interference peaks were observed at the retention times corresponding to each compound in the blank chromatogram. From top to bottom: Bupleurum saponin D (m/z 779.6 → 617.4, negative ion), glycyrrhizin (m/z 417.2 → 255.0, negative ion), and paeonol glycoside (m/z 481.4 → 197.2, positive ion). The retention times are 4.64 min for Bupleurum saponin D, 2.94 min for glycyrrhizin, and 2.77 min for paeonol glycoside.

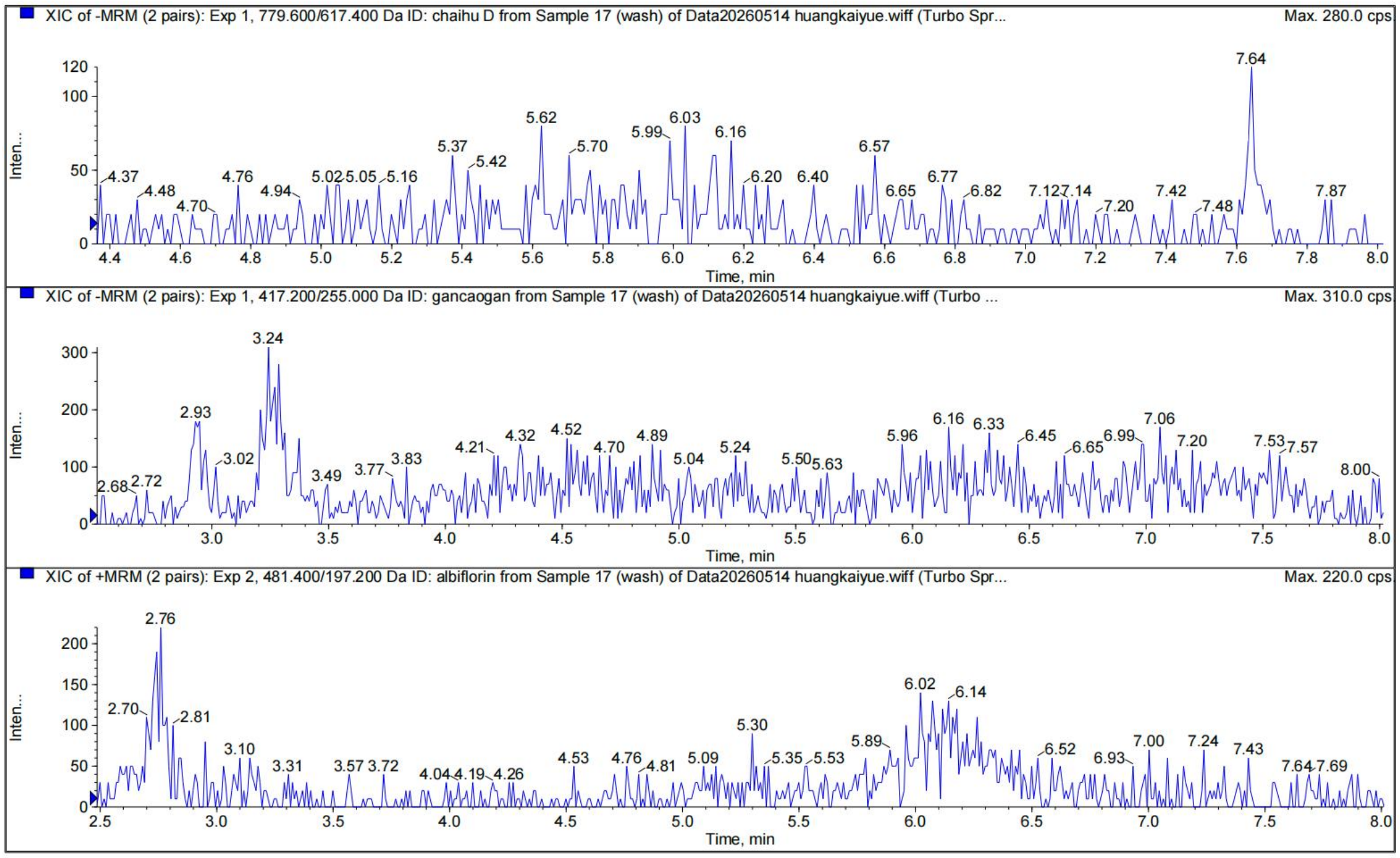

Figure S6(A) Blank control. From top to bottom: Bapgenin (m/z 779.6→617.4); Glycyrrhizin (m/z 417.2→255.0); Paeonol (m/z 481.4→197.2).

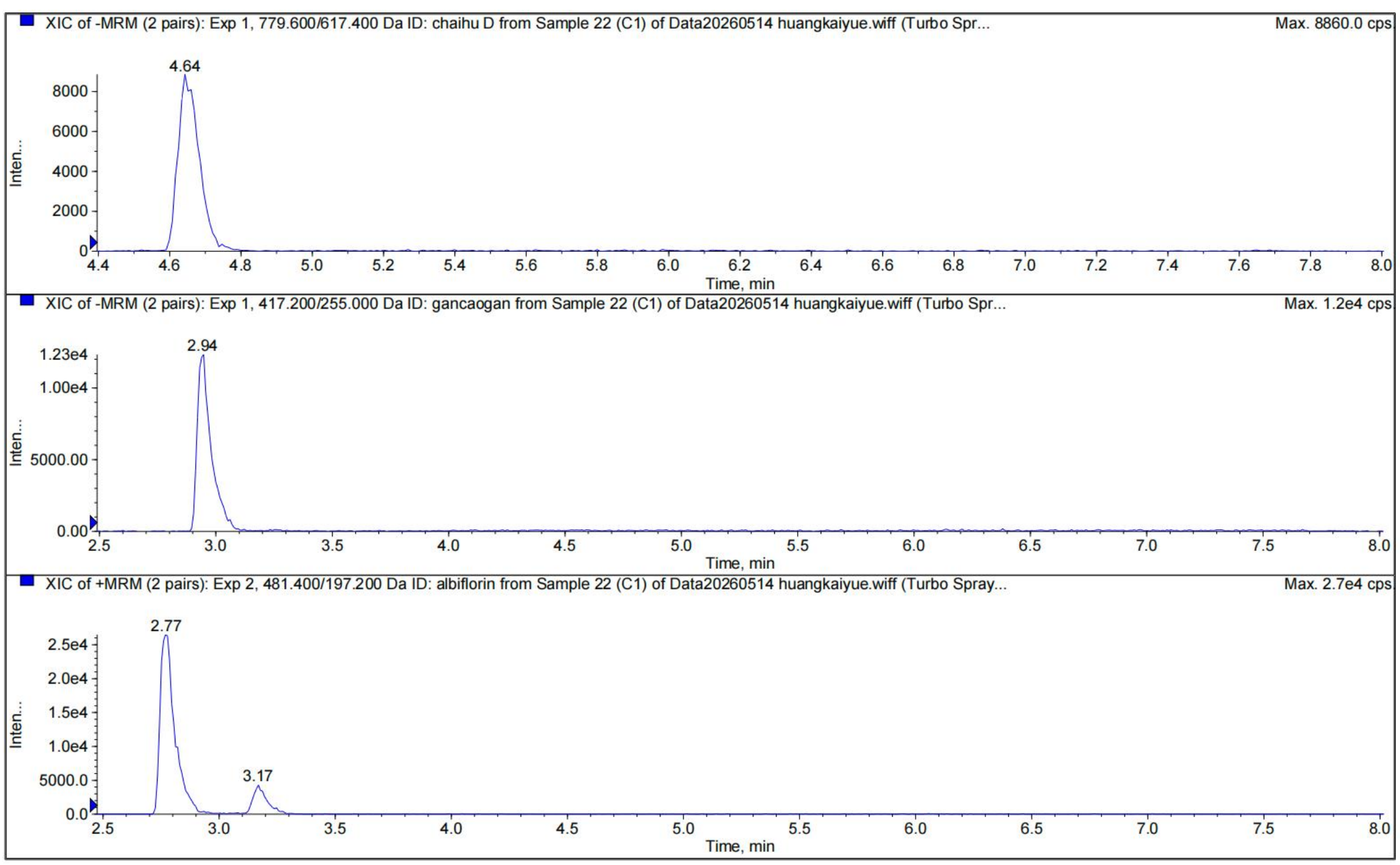

Figure S6(B) Shows samples at the limit of quantification (LLOQ). From top to bottom: Bapgenin (m/z 779.6 → 617.4); Glycyrrhizin (m/z 417.2 → 255.0); Paeonol glycoside (m/z 481.4 → 197.2).

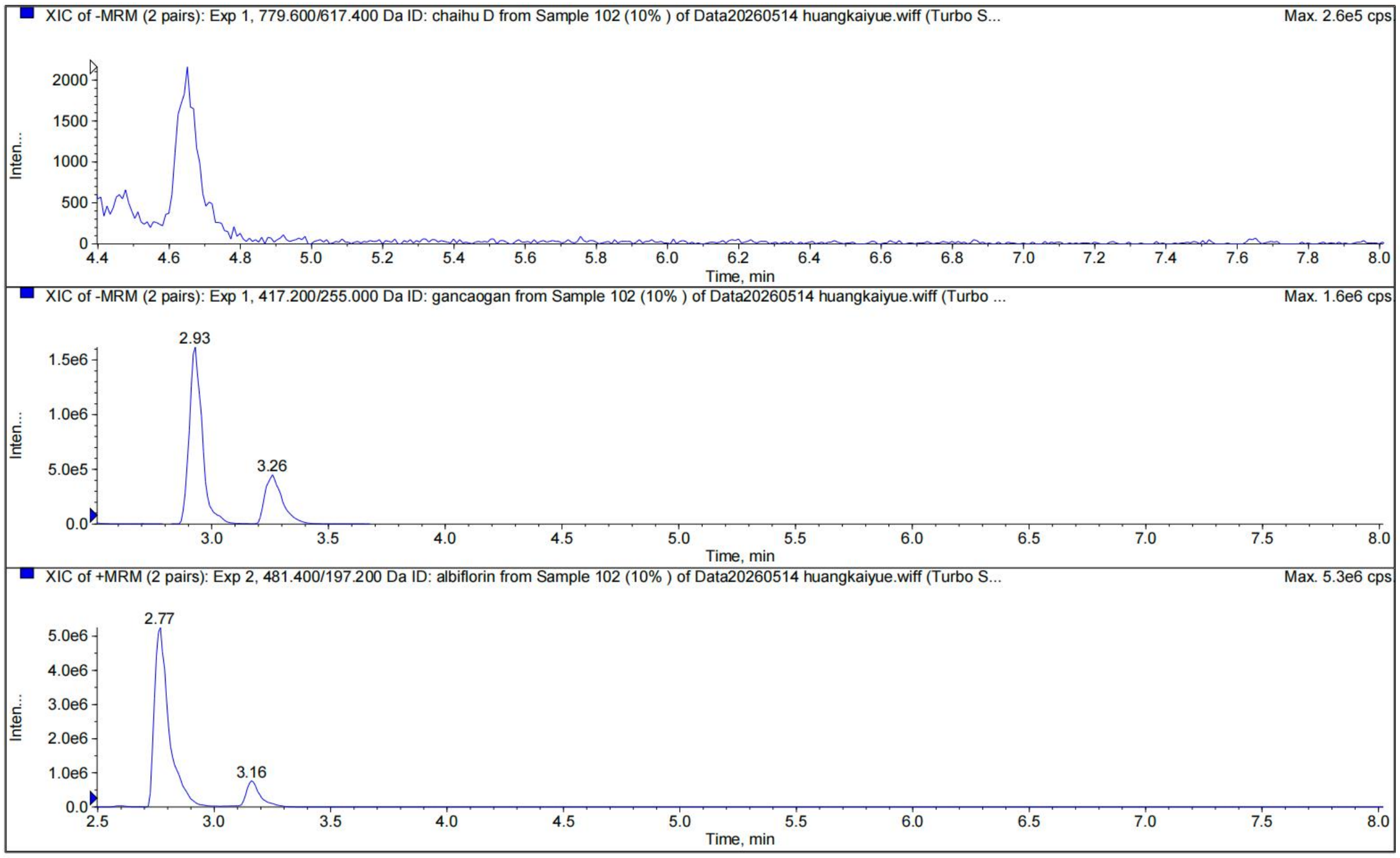

Figure S6(C) Test solution (CSS). From top to bottom: Bupleurum saponin D (m/z 779.6→617.4); glycyrrhizin (m/z 417.2→255.0); paeonol glycoside (m/z 481.4→197.2).
